# 5-Ene-2-arylaminothiazol-4(5*H*)-ones Induce Apoptosis in Breast Cancer Cells

**DOI:** 10.3390/cells14120861

**Published:** 2025-06-07

**Authors:** Rostyslav Dudchak, Magdalena Podolak, Ivan Sydorenko, Robert Czarnomysy, Agnieszka Gornowicz, Olexandr Karpenko, Serhii Holota, Anna Bielawska, Krzysztof Bielawski, Roman Lesyk

**Affiliations:** 1 Department of Synthesis and Technology of Drugs, Faculty of Pharmacy, Medical University of Bialystok, Jana Kilińskiego 1, 15-089 Bialystok, Poland; robert.czarnomysy@umb.edu.pl (R.C.); krzysztof.bielawski@umb.edu.pl (K.B.); 2 Department of Biotechnology, Faculty of Pharmacy, Medical University of Bialystok, Jana Kilińskiego 1, 15-089 Bialystok, Poland; magdalena.podolak@sd.umb.edu.pl (M.P.); agnieszka.gornowicz@umb.edu.pl (A.G.); anna.bielawska@umb.edu.pl (A.B.); 3 Department of Pharmaceutical, Organic and Bioorganic Chemistry, Danylo Halytsky Lviv National Medical University, Pekarska 69, 79010 Lviv, Ukraine; alterivan12@gmail.com (I.S.); golota_serg@yahoo.com (S.H.); 4 Department of Chemistry, Taras Shevchenko National University of Kyiv, 64 Volodymyrska St., 01033 Kyiv, Ukraine; karalexander@gmail.com; 5 Enamine Ltd., Winston Churchill 78, 02094 Kyiv, Ukraine; 6 Molecular Design Center, Danylo Halytsky Lviv National Medical University, Pekarska 69, 79010 Lviv, Ukraine; 7 Department of Biotechnology and Cell Biology, University of Information Technology and Management in Rzeszow, St. Sucharskiego 2, 35-225 Rzeszow, Poland

**Keywords:** anticancer, 4-thiazolidinone, breast cancer, apoptosis

## Abstract

As breast cancer remains a significant challenge for the current medical field, molecules with a 4-thiazolidinone scaffold can become promising candidates for addressing the increasing threat of cancer. This study aims to develop and evaluate the novel 4-thiazolidinone derivatives with anticancer potential. New compounds were synthesized through two different pathways, one as a two-step process and the other as a one-pot method. The second approach fits the requirements of cost-effective methodologies and allows for the reduction of synthetic steps, reagents, and reaction time. The obtained data from in vitro research showed a potent cytotoxic activity of the novel structures in micromolar concentrations against MCF-7 breast cancer cells. Further investigations into their anticancer activity revealed that the tested compounds induced apoptosis through intrinsic and extrinsic pathways, which was evidenced by their capability to reduce the mitochondrial membrane potential and induce the activation of caspases 7, 8, 9, and 10. A more detailed analysis uncovered that one of the novel compounds can affect the expression of key apoptotic proteins, tumor protein P53 (p53), cytochrome C, and Bax in treated cells. Additionally, these compounds displayed an enhanced generation of reactive oxygen species (ROS) in MCF-7 cells, which suggests that ROS-mediated mechanisms can take part in the anticancer potential of the synthesized compounds.

## 1. Introduction

The persistent threat posed by cancer continues to be one of the primary concerns for global society. According to the American Cancer Society, 2,001,140 new cancer cases and 611,720 cancer deaths are predicted to happen in the United States in 2024. Among them, 313,510 are estimated to be new breast cancer cases, with 42,780 projected deaths from this type of cancer [1]. Those numbers are especially concerning, taking into consideration the incidence rate, which has consistently risen for the last four decades with a 0.5% annual increase in the years 2010–2019 [2]. While chemotherapy remains the main method of treatment for breast cancer, the potential long-term toxicities may affect a great number of patients. A spectrum of adverse effects, including but not limited to fatigue, insomnia, peripheral neuropathy, cognitive impairment, estrogen deprivation, cardiotoxicity, and second cancers are capable significantly compromise the quality of life for breast cancer patients [3].

4-Thiazolidinones present promising scaffolds for the design of potential anticancer agents [4,5]. Applying the hybrid-pharmacophore approach is one of the leading strategies for the research and development of novel molecules with satisfactory antitumor properties and toxicology parameters among these heterocycles [6,7]. Substituted 2-arylamino-4-thiazolidones are among the most often used parent molecules in the hybridization process for synthesizing anticancer hit and lead compounds [8,9,10]. The pharmacological significance and importance of the mentioned scaffolds are emphasized by the fact that there are numerous 4-thiazolidinone-containing drugs approved by the FDA. For example, antihyperglycemic drugs (Rosiglitazone [11] and Pioglitazone [12]), aldose reductase inhibitors (Epalrestat [13]), anti-inflammatory drugs (Darbufelon [14]), diuretics (Etozoline [15]), anticonvulsants (Ralitolin [16]) and drugs for treatment of multiple sclerosis (Ponesimod [17]). From a molecular mechanistic point of view, 4-thiazolidinone-bearing hybrids represent a promising direction to cancer treatment, leveraging multiple mechanisms of action to target various aspects of cancer cells like survival, proliferation, and apoptosis. Apoptosis, known as programmed cell death, is a crucial physiological process vital for proper development and the maintenance of tissue homeostasis. It can proceed via two main pathways: extrinsic (death receptor-mediated) and intrinsic (mitochondria-mediated) [18]. 4-Thiazolidinone derivatives have been shown to induce apoptosis across a wide range of cancer cell lines [19,20]. Furthermore, among 4-thizolidinone derivatives, there are numerous examples of their anticancer effects through targeting various molecular targets such as PPARγ [21], human topoisomerase [22], β-tubulin [23,24,25], or through ROS generation [26].

This study aimed to develop and synthesize 4-thiazolidinone derivatives with potential anticancer activity, with a particular focus on their capacity to induce apoptosis through both pathways and related proteins.

## 2. Materials and Methods

### 2.1. Chemistry

To determine melting points, open capillary tubes on a BÜCHI B-545 melting point apparatus (BÜCHI Labortechnik AG, Flawil, Switzerland) were utilized. The elemental analyses (C, H, and N) were performed using the Perkin-Elmer 2400 CHN analyzer (PerkinElmer, Waltham, MA, USA) and were within ± 0.4% of the theoretical values. The 500 MHz ^1^H and 126 MHz ^13^C spectra were recorded on a Bruker AVANCE-500 spectrometer (Bruker, Bremen, Germany). Obtained spectra were documented at room temperature, except where it was indicated differently, and were referenced internally to solvent reference frequencies. Chemical shifts (δ) are quoted in ppm, and coupling constants (*J*) are reported in Hz. LC–MS spectra were determined on a Finnigan MAT INCOS-50 (Thermo Finnigan LLC, San Jose, CA, USA). Commercially available solvents and reagents were utilized without further purification. Starting 2-chloro-N-(3,4,5-trimethoxyphenyl)acetamide (**1**) was obtained from commercially available 3,4,5-trimethoxyaniline (CAS 24313-88-0) and chloroacetyl chloride (CAS 79-04-9) accordingly to the protocol reported in [27].

*2-((3,4,5-Trimethoxyphenyl)amino)thiazol-4(5H)-one (2)*

The mixture of 2-chloro-N-(3,4,5-trimethoxyphenyl)acetamide (**1**) (10 mmol) and ammonium thiocyanate (20 mmol) in acetone (10 mL) was heated to reflux for 6 h. The progress of the reaction was monitored by TLC. After completion, the reaction mixture was cooled to room temperature, and the solvent was evaporated. The resultant solid of compound **2** was washed with water, methanol (5–10 mL), diethyl ether, and crystallized from the mixture of DMF–ethanol (1:3).

Beige powder, yield 64%, mp 206–208 °C (DMF–ethanol). ^1^H NMR (500 MHz, DMSO-*d*_6_, δ): 11.70 (br.s, 1H, NH), 11.07 (s, 1H, NH), 7.06 (br.s, 1H, arom.), 6.28 (s, 1H, arom.), 3.97 and 3.94 (2*s, 2H, CH_2_), 3.74 and 3.73 (2*s, 9H, 3*CH_3_). LCMS (ESI+) *m/z* 283.0 (95.0%, [M + H]^+^). Anal. calc. for C_12_H_14_N_2_O_4_S: C, 51.05%; H, 5.00%; N, 9.92%. Found: 51.20%; H, 5.20%; N, 10.10%.

*Synthesis of 5-ene-2-((3,4,5-trimethoxyphenyl)amino)thiazol-4(5H)-ones Les-6381, Les-6416, Les-6418, Les-6423, and Les-6424*

The mixture of 2-((3,4,5-trimethoxyphenyl)amino)thiazol-4(5H)-one (**2**) (10 mmol), the appropriate aromatic/heterocyclic aldehyde (12.5 mmol), and anhydrous sodium acetate (10 mmol) in glacial acetic acid (10 mL) was heated to reflux for 3–4 h. The progress of the reaction was monitored by TLC. After completion, the reaction mixture was cooled to room temperature. The resultant solids of target compounds were washed with water, methanol, diethyl ether, and crystallized from the mixture of DMF–ethanol (1:3).

*Multicomponent protocol*

The mixture of 2-chloro-N-(3,4,5-trimethoxyphenyl)acetamide (**1**) (10 mmol), ammonium thiocyanate (12.5 mmol) appropriate aromatic/heterocyclic aldehyde (10 mmol), and ethylenediamine diacetate (EDDA) (10 µmol) in 2-propanol (10 mL) was heated to reflux for 3 h. The progress of the reaction was monitored by thin-layer chromatography (TLC) using commercial glass-backed TLC plates (Merck Kieselgel 60 F254, Merck, Darmstadt, Germany). After completion, the reaction mixture was cooled to room temperature. The resultant solids of target compounds were washed with water, methanol, diethyl ether, and crystallized from the mixture of DMF–ethanol (1:3).

*5-(4-Methoxybenzylidene)-2-((3,4,5-trimethoxyphenyl)amino)thiazol-4(5H)-one (Les-6381)*

White powder, yield 66% (A), 57% (B), mp 222–224 °C (DMF–EtOH). ^1^H NMR (500 MHz, DMSO-*d*_6_, δ): ^1^H NMR (500 MHz, DMSO) δ 9.73 (s, 1H, NH), 7.64 (s, 1H, CH=), 7.54 (d, *J* = 8.2 Hz, 2H, arom.), 7.11 (d, *J* = 8.3 Hz, 2H, arom.), 6.71 (s, 2H, arom.), 3.83 (s, 3H, CH_3_), 3.75 (s, 6H, 2*CH_3_), 3.71 (s, 3H, CH_3_). ^13^C NMR (126 MHz, DMSO-*d*_6_, δ): 167.9 (C=O), 152.9, 137.5, 133.7, 131.6, 128.2, 126.2, 123.0, 114.8, 111.3, 110.9, 106.7, 59.9 (CH_3_), 56.1 (CH_3_), 55.4 (CH_3_), 45.1 (CH_3_). LCMS (ESI+) *m/z* 401.0 (98.9%, [M + H]^+^). Anal. calc. for C_20_H_20_N_2_O_5_S: C, 59.99%; H, 5.03%; N, 7.00%. Found: C, 60.20%; H, 5.20%; N, 7.10%.

*5-((1H-Indol-6-yl)methylene)-2-((3,4,5-trimethoxyphenyl)amino)thiazol-4(5H)-one (Les-6416)*

Yellow powder, yield 58% (A), 64% (B), mp > 250 °C (DMF–EtOH). ^1^H NMR (500 MHz, DMSO-*d*_6_, δ): 11.49 and 11.39 (br.s, 1H, NH), 9.72 (br.s, 1H, NH), 7.80 s, 7.73 br.s, 7.68 d (*J* = 8.5 Hz), 7.64–7.57 m, 7.54 br.s, 7.49 s, 7.24 d (*J* = 8.4 Hz), 7.21–7.14 m, 6.73 s, 6.51 s, 6.37 s (8H, arom.+CH=); 3.79–3.73 m, 3.71 s, 3.67 s, (9H, 3*CH_3_). ^13^C NMR (126 MHz, DMSO-*d*_6_, δ): 166.4 (C=O), 152.9, 138.3, 135.9, 134.9, 130.6, 130.4, 129.0, 128.6, 125.5, 121.1, 120.7, 119.4, 119.0, 113.4, 106.6, 101.6, 59.9 (CH_3_), 56.1 (2*CH_3_). LCMS (ESI+) *m/z* 410.0 (73.0/25.7%, [M + H]^+^). Anal. calc. for C_21_H_19_N_3_O_4_S: C, 61.60%; H, 4.68%; N, 10.26%. Found: 61.70%; H, 4.80%; N, 10.30%.

*5-((1H-Indol-3-yl)methylene)-2-((3,4,5-trimethoxyphenyl)amino)thiazol-4(5H)-one (Les-6418)*

Yellow powder, yield 58% (A), 55% (B), mp > 250 °C (DMF–EtOH). ^1^H NMR (500 MHz, DMSO-*d*_6_, δ): 11.94 (s, 1H, NH), 11.83 (s, 1H, NH), 7.96 and 7.88 (2*s, 1H, =CH), 7.82 (d, *J* = 7.9 Hz, 1H, arom.), 7.67 and 7.63 (2*s, 1H, arom.), 7.47 (d, *J* = 8.2 Hz, 1H, arom.), 7.18 (dt, *J* = 28.2, 7.4 Hz, 2H, arom.), 6.36 (s, 2H, arom.), 3.77 (s, 6H, 2*CH_3_), 3.66 (s, 3H, CH_3_). ^13^C NMR (100 MHz, DMSO-*d*_6_, δ): 165.7, 152.9, 137.6, 136.1, 131.6, 130.8, 127.7, 126.6, 122.9, 120.8, 120.7, 118.3, 116.1, 112.2, 110.8, 106.7, 59.9, 56.1. LCMS (ESI+) *m/z* 410.0 (100.0%, [M + H]^+^). Anal. calc. for C_21_H_19_N_3_O_4_S: C, 61.60%; H, 4.68%; N, 10.26%. Found: 61.80%; H, 4.90%; N, 10.40%.

*5-(Thiophen-2-ylmethylene)-2-((3,4,5-trimethoxyphenyl)amino)thiazol-4(5H)-one (Les-6423)*

Yellow powder, yield 67% (A), 64% (B), mp > 250 °C (DMF–EtOH). ^1^H NMR (500 MHz, DMSO-*d*_6_, δ): 12.05 (s, 1H, NH); 7.94 (d, *J* = 11.2 Hz, 1H, arom.), 7.88 (s, 1H, CH=); 7.62 and 7.57 (2*br.s, 1H, arom.), 7.27 and 7.22 (2*br.s, 1H, arom.), 7.12 (br.s, 1H, arom.), 6.38 (s, 1H, arom.), 3.77 (br.s, 6H, 2*CH_3_), 3.66 (br.s, 3H, CH_3_). ^13^C NMR (126 MHz, DMSO-*d*_6_, δ): 164.6 (C=O), 158.7, 153.8 and 153.4, 139.1, 137.7, 133.8, 132.4, 131.5, 129.1, 123.9, 123.1, 106.0, 99.4 and 98.9, 60.5 (CH_3_), 56.4 (2*CH_3_). LCMS (ESI+) *m/z* 377.0 (98.6%, [M + H]^+^). Anal. calc. for C_17_H_16_N_2_O_4_S_2_: C, 54.24%; H, 4.28%; N, 7.44%. Found: C, 54.40%; H, 4.40%; N, 7.60%.

*5-(Furan-2-ylmethylene)-2-((3,4,5-trimethoxyphenyl)amino)thiazol-4(5H)-one (Les-6424)*

Yellow powder, yield 60% (A), 62% (B), mp > 250 °C (DMF–EtOH). ^1^H NMR (500 MHz, DMSO-*d*_6_, δ): 12.00 (br.s, 1H, NH); 8.01 and 7.95 (2*s, 1H, arom.), 7.51 and 7.43 (2*s, 1H, CH=), 7.12 (s, 1H, arom.), 7.00 and 6.96 (2*s, 1H, arom.), 6.73 and 6.67 (2*s, 1H, arom.), 6.34 (s, 1H, arom), 3.78 and 3.75 (2*s, 6H, 2*CH_3_), 3.65 (s, 3H, CH_3_). ^13^C NMR (126 MHz, DMSO-*d*_6_, δ): 165.6 (C=O), 157.9, 153.7 and 153.2, 150.0, 147.1, 134.9, 124.3, 121.2, 120.3, 117.5, 116.5, 113.7, 99.4 and 99.0, 60.5 (CH_3_), 56.4 (2*CH_3_). LCMS (ESI+) *m/z* 361.0 (100.0%, [M + H]^+^). Anal. calc. for C_17_H_16_N_2_O_5_S: C, 56.66%; H, 4.48%; N, 7.77%. Found: C, 56.80%; H, 4.60%; N, 7.90%.

Doxorubicin hydrochloride (DOX) was obtained from Sigma-Aldrich (St. Louis, MO, USA).

### 2.2. NCI Anticancer Screening In Vitro

The primary anticancer assay was performed on a panel of sixty human cancer cell lines derived from nine diseases, in accordance with the protocol of the Drug Evaluation Branch, National Cancer Institute, Bethesda [28,29,30]. Novel compounds were added to the cells at a single concentration (10^−2^ µM) and incubated for 48 h. The cytotoxic and/or growth inhibitory effects of every compound were evaluated using the sulforhodamine B (SRB) assay. The obtained data were reported as the percentage of growth of the treated cells compared to the untreated control. The most active compounds were tested in vitro against the full panel of human tumor cell lines at concentrations in the range between 10^−4^ and 10^−8^ M for 48 h. The SRB assay was used to estimate cell viability or growth. Using absorbance measurements [time zero (Tz), control growth in the absence of drug (C), and test growth in the presence of drug (Ti)], the percentage growth was calculated for each drug concentration. Percentage growth inhibition was calculated as follows:

[(Ti − Tz)/(C − Tz)] × 100 for concentrations for which Ti ≥ Tz,

[(Ti − Tz)/Tz] × 100 for concentrations for which Ti < Tz.

Dose–response parameters (GI_50_, TGI) were calculated for each compound. Molar concentration of the compound that inhibits 50% net cell growth (GI_50_) was calculated from [(Ti − Tz)/(C − Tz)] × 100 = 50, which is the drug concentration resulting in a 50% lower net protein increase in the treated cells (measured by SRB staining) as compared to the net protein increase seen in the control cells. The drug concentration resulting in total growth inhibition (TGI) was calculated from Ti = Tz. Values were calculated for each of these parameters if the level of activity was reached; however, if the effect was not reached or was excessive, the value for that parameter was expressed as more or less than the maximum or minimum concentration tested. Compounds having GI_50_ values ≤ 100 μM were declared to be active.

### 2.3. Cell Culture

All of the tested cell lines were acquired from the American Type Culture Collection (ATCC, Manassas, VA, USA), including human breast cancer cell lines (MCF-7, MDA-MB-231), human gastric cancer cell line (AGS), human colorectal cancer cell lines (HT-29, DLD-1), human glioblastoma cancer cell lines (T98G, A172), mouse astrocyte type I clone (C8-D1A), human normal epithelial breast cells (MCF-10A) and primary dermal fibroblast (HDFa). Such cell lines as AGS, MCF-7, MDA-MB-231, A172, T98G, C8-D1A, and primary dermal fibroblast (HDFa) were cultured in complete medium–DMEM (Corning, Kennebunk, ME, USA). RPMI-1640 (ATCC, Manassas, VA, USA) was used for the DLD-1 cell line, and McCoy’s 5A (PAN-Biotech, Aidenbach, Germany) for the HT-29 cell line. To all utilized media additionally was added 10% fetal bovine serum (FBS) (Eurx, Gdansk, Poland) and 1% penicillin and streptomycin solution (Corning, Kennebunk, ME, USA). MCF-10A cells were incubated in MEGM (Mammary Epithelial Cell Growth Medium) BulletKit supplemented with penicillin/streptomycin cocktail and 5% horse serum. All cells were cultured at 37 °C in an atmosphere with 5% CO₂ in tissue culture dishes (Sarstedt, Numbrecht, Germany). For experiments, cells were washed with phosphate-buffered saline (PBS) (Corning, Kennebunk, ME, USA) followed by the addition of 0.05% trypsin with 0.02% EDTA (Corning, Kennebunk, ME, USA). Scepter 3.0 cell counter (Merck Millipore, Burlington, MA, USA) was applied before each seeding to calculate the number of cells. For the next analyses, cells were reseeded in 6-well plates, 24-well plates, and 96-well plates.

### 2.4. Cell Viability Assay

MTT (3-(4,5-dimethylthiazol-2-yl)-2,5-diphenyltetrazolium bromide) assay method was utilized to analyze compound effects on cell viability. All cell lines were seeded in 96-well plates with 1 × 10^4^ cells per well. After 24 h, cells were treated with the tested compounds and reference drug at concentrations of 0.1, 1, 2.5, 5, 10, and 20 µM for 24, 48, or 72 h. Next, a solution of 3-(4,5-dimethyl-2-thiazolyl)-2,5-diphenyl-2H-tetrazolium bromide (Sigma-Aldrich, St. Louis, MO, USA) in PBS with a concentration of 5 mg/mL was prepared. After the incubation with tested compounds, 10 µL of the prepared MTT solution was applied to each well, followed by 2 h of incubation at 37 °C. Next, cells underwent lysis by adding 100 µL of pre-prepared lysing buffer (containing 25 mM HCl, 2% acetic acid, 3% DMF, and 5% SDS, with a pH of 4.7). The absorbance was measured using a UV-VIS Helios Gamma (Unicam/ThermoFisher Scientific Inc., Cleveland, OH, USA) at 570 nm wavelength. The cells incubated with vehicle substance (0.1% DMSO) were taken as controls.

### 2.5. [^3^H]-Thymidine Incorporation Assay

To evaluate the effects of synthesized compounds and reference drug on cell proliferation [3H]-thymidine incorporation assay was applied [31]. MCF-7 cells were seeded on 6-well plates as described before. After 24 h of incubation, the cells were treated with different concentrations of novel 4-thiazolidinone derivatives Les-6416, Les-6418 (1, 2.5, 5 µM), and doxorubicin hydrochloride (0.5 and 1 µM) for 24 h at 37 °C. After 24 h, the medium was changed to a fresh one without fetal bovine serum. A total of 0.5 µCi of tritium-labeled thymidine (specific activity: 7 Ci/mmol) was added to cells for 4 h. Afterwards, the cells were washed with 1 mL of 0.05 M Tris-HCl buffer pH 7.4 containing 0.11 M NaCl and 1 M 5% trichloroacetic acid (TCA). Each well was filled with 1 mL of a 0.1 M NaOH solution with 1% SDS for cell lysis. After 5 min, the cell lysates were transferred to scintillation vials, which were pre-filled beforehand with two milliliters of scintillation cocktail Ultima Gold XR from PerkinElmer (Waltham, MA, USA). The Scintillation Counter 1900 TR, TRI-CARB (Packard, Perkin Elmer, Inc., San Jose, CA, USA) was used to record the results. The radioactivity observed in untreated control cells was taken as 100%. The values of the tested compound were expressed as a percentage of the control value.

### 2.6. Clonogenic Assay

The clonogenic assay was performed in accordance with the published methodology [32]. MCF-7 cells were seeded in 24-well plates at 2.5 × 10^2^ cells per well in 1 mL medium. Afterwards, they were left in an incubator at 37 °C for 24 h to attach to the wells. On the next day, the tested compounds Les-6416, Les-6418 (1, 2.5, 5 µM), and doxorubicin hydrochloride (0.5 and 1 µM) were added to the wells for 24 h treatment. After the treatment, the medium was changed to a fresh one and left for 10 days, with an additional change in medium for the fresh one on the 7th day. Next, colonies formed by the seeded cells were washed, fixed for 10 min at 4 °C with 90% methanol, and stained with crystal violet solution (0.1% of crystal violet dissolved in 25% methanol). Afterwards, the plates were washed and dried at room temperature. The wells were photographed and analyzed using ImageJ software version 1.54k (Bethesda, MD, USA), with colonies defined as clusters of fifty or more cells [33].

### 2.7. Scratch Assay

To assess the effects of the tested compounds on cell migration, the scratch assay method was applied [34]. Cells were seeded in 24-well plates at 2 × 10^5^ cells per well in 1 mL medium and incubated overnight. Afterwards, they were left in an incubator at 37 °C for 24 h to attach to the wells. The next day, scratches were made using a 200 µL pipette tip in confluent monolayers. Afterwards, the wells were washed to remove the debris, and tested compounds (Les-6416, Les-6418) were added in concentrations: 1, 2.5, and 5 µM, while the reference drug–doxorubicin hydrochloride was added in concentrations: 0.5 and 1 µM. Images were obtained by utilizing a Nikon ECLIPSE Ti microscope (Nikon, Tokyo, Japan) after 0, 24, and 48 h. The area of the scratches was measured manually via ImageJ software version 1.54k (Bethesda, MD, USA).

### 2.8. Annexin V Binding Assay

To assess the induction of apoptosis as a result of novel compounds’ effects FITC Annexin V Apoptosis Detection Kit II (BD Biosciences, San Diego, CA, USA) was utilized as described previously [35]. The MCF-7 cells were seeded on 6-well plates (1 × 10^5^ cells per well) and incubated for 24 h. Afterwards, the cells were washed, and tested compounds (Les-6416, Les-6418, Les-6381, Les-6423, and Les-6424) were added in a concentration of 5 µM. The reference drug doxorubicin hydrochloride was applied in a concentration of 0.5 µM. The cells were incubated with the compounds for 24 h. Afterwards, the cells were washed several times with cold PBS and the cell suspensions were prepared in the binding buffer from the kit at a concentration of 1 × 10^6^ cells/mL. A total of 100 µL of suspension was collected from each sample and transferred to the tubes. After that, 5 µL of Annexin V-FITC and 5 µL of propidium iodide were added. After 15 min of incubation at room temperature in a dark environment, the content of the tubes was filled with binding buffer up to 500 µL and assessed by BD FACSCanto II flow cytometer (Becton Dickinson Biosciences, San Jose, CA, USA). A total of 1 × 10^4^ events were measured for each sample. The results were investigated by applying FACSDiva software Version 6.1.3. (BD Biosciences Systems, San Jose, CA, USA).

### 2.9. Caspases-3/7, Caspase-8, Caspase-9, and Caspase-10 Activity Assessment

To determine the effects of the novel compounds on the caspases-3/7, caspase-8, caspase-9, and caspase-10 activity, the FLICA Caspase-3/7 Assay Kit, FLICA Caspase-8 Assay Kit, FLICA Caspase-9 Assay Kit, and FLICA Caspase-10 Assay Kit were applied (ImmunoChemistry Technologies, Bloomington, MN, USA) [35]. MCF-7 cells were seeded on 6-well plates (1 × 10^5^ cells per well) and incubated for 24 h. Afterwards, they were treated for 24 h with tested compounds (Les-6416, Les-6418, Les-6381, Les-6423, and Les-6424) in a concentration of 5 µM and the reference drug doxorubicin hydrochloride in a concentration of 0.5 µM. Next, cells were amassed and washed two times with cold PBS, followed by resuspension in an Apoptosis Wash Buffer to a final concentration of 5 × 10^5^ cells/mL. Afterwards, 290 µL of the prepared cell suspensions were transferred into tubes, followed by the addition of 10 µL of FLICA solution, prepared immediately before use, to each sample and incubated for an hour at 37 °C. Next, cells were washed two times with 2 mL of Apoptosis Wash Buffer, provided in the kit, centrifuged, and resuspended in 300 µL of the buffer. Prepared samples were assessed by a BD FACSCanto II flow cytometer (Becton Dickinson Biosciences, San Jose, CA, USA). For each sample, 1 × 10^4^ events were analyzed. The obtained results were investigated by applying FACSDiva software Version 6.1.3 (BD Biosciences Systems, San Jose, CA, USA).

### 2.10. Assessment of the Decrease in Mitochondrial Membrane Potential (MMP)

Lipophilic fluorochrome JC-1 (5,5′,6,6′-tetrachloro-1,1′,3,3′-tetraethylbenzimidazolcarbocyanine iodide) (BD Biosciences, San Diego, CA, USA) was applied to determine the novel structures’ effects on the MMP [35]. MCF-7 cells were seeded on 6-well plates at 5 × 10^6^ cells per well, followed by incubation for 24 h. Afterwards, the cells were treated for 24 h with new 4-thiazolidinone derivatives (Les-6416, Les-6418, Les-6381, Les-6423, and Les-6424) in a concentration of 5 µM and the reference drug doxorubicin hydrochloride in a concentration of 0.5 µM. Then, cells were collected at a concentration of 1 × 10^6^ cells/mL and washed two times with PBS. Next, 0.5 mL of the pre-prepared JC-1 working solution was added to each sample in accordance with the instructions of the manufacturer, followed by incubation at 37 °C for 15 min in a CO_2_ incubator. After that, an assay buffer was added to each sample to wash the cells several times. Afterwards, 0.5 mL from each sample was transferred to the tubes for immediate assessment using a BD FACSCanto II flow cytometer (Becton Dickinson Biosciences, San Jose, CA, USA). For each sample, 1 × 10^4^ events were analyzed. The obtained results were investigated by applying FACSDiva software Version 6.1.3 (BD Biosciences Systems, San Jose, CA, USA).

### 2.11. Assessment of p53 and Cytochrome C Concentration

To determine the effects of the new 4-thiazolidinone derivatives and the reference drug on the concentration of p53 and cytochrome C in MCF-7 cell lysates or supernatants after 24 h of treatment, highly sensitive SimpleStep ELISA (enzyme-linked immunosorbent assay) kits (Abcam, Cambridge, UK) were applied [36]. The cells were collected and washed thrice with cold PBS and centrifuged at 1000× *g* for 5 min at 4 °C. Next, the cells were suspended in lysis buffer to acquire cell lysates. Concurrently, the supernatants, acquired after centrifugation, were frozen immediately at −80 °C. The standards, samples, and antibody cocktail were added to the appropriate microtiter plate wells, followed by 1 h incubation at room temperature. The content of microplate wells was extracted, and the wells were washed thrice. Afterwards, a TMB development solution was added to each well. The enzyme–substrate reaction was terminated with a stop solution. The change in color was assessed by UV-VIS Helios Gamma (Unicam/ThermoFisher Scientific Inc., Cleveland, OH, USA) at 450 ± 2 nm wavelength.

### 2.12. Assessment of Bax Concentration

To evaluate the effects of the tested compounds on the concentration of Bax protein, anti-Bax FITC-conjugated antibody was applied. MCF-7 cells were seeded on 6-well plates at 5 × 10^6^ cells per well, followed by incubation for 24 h. Afterwards, the cells were treated for 24 h with new 4-thiazolidinone derivatives (Les-6416, Les-6418, Les-6381, Les-6423, and Les-6424) at a concentration of 5 µM. Then, cells were collected and incubated with 4% formaldehyde for 15 min at RT. Next, cells were washed with an excess amount of PBS, followed by permeabilization by adding ice-cold 90% methanol and incubation for 60 min in an ice bath. Cells were again washed with an excess of PBS and resuspended in 100 µL of diluted primary antibody, prepared in antibody dilution buffer at a 1:100 dilution, and incubated for 60 min at room temperature in the dark. Cells were washed, resuspended in 0.3 mL, and transferred to the tubes for immediate assessment using a BD FACSCanto II flow cytometer (Becton Dickinson Biosciences, San Jose, CA, USA). For each sample, 1 × 10^4^ events were analyzed. The results were analyzed using FACSDiva software Version 6.1.3 (BD Biosciences Systems, San Jose, CA, USA).

### 2.13. Reactive Oxygen Species Assessment

To evaluate the effects of the most potent tested compound Les-6416 (2.5, 5 µM) and the reference drug doxorubicin hydrochloride (0.5 and 1 µM) on ROS generation a double staining method with 2′,7′-dichlorodihydrofluorescein diacetate (H_2_DCFDA) reagent (Thermo Fisher Scientific, Eugene, OR, USA) and fluorescent dye 4′,6-diamidino-2-phenylindole (DAPI) (Sigma-Aldrich, St. Louis, MO, USA) was applied. MCF-7 cells were seeded in 6-well plates at 1 × 10^5^ cells per well in 2 mL medium. Afterwards, cells were treated with the mentioned compounds for 1 and 3 h, respectively. Next, cells were washed, and 20 µM of H_2_DCFDA was added for 30 min. After that, 10 µg/mL of DAPI was added to observe the nuclear DNA of cells. The results were obtained with the use of a Nikon ECLIPSE Ti fluorescence microscope (Nikon, Tokyo, Japan), followed by further assessment via ImageJ software version 1.54k (Bethesda, MD, USA).

### 2.14. Morphological Analysis of Cells by Acridine Orange (AO) and Ethidium Bromide (EB) Staining

To display morphological changes in the MCF-7 cells after 24 h of treatment by lead compound Les-6416 and the positive control doxorubicin, we performed acridine orange and ethidium bromide staining. MCF-7 cells were seeded in 6-well plates at 1 *×* 10^5^ cells per well in 2 mL medium, followed by incubation for 24 h. Afterwards, the cells were treated with lead compound Les-6416 at a concentration equal to 5 µM and DOX in *a* concentration equal to 0.5 µM. Next, cells were washed with 1 mL of PBS per well. Subsequently, double staining was performed using 10 µL of AO and EB, incubated at room temperature for 10 min. After that, cells were washed with PBS. Images were obtained with the use of *a* Nikon ECLIPSE Ti fluorescence microscope (Nikon, Tokyo, Japan). Images were analyzed using ImageJ software version 1.54k (Bethesda, MD, USA).

### 2.15. In Silico ADMET Evaluation

ADMETLab 3.0 software was utilized to evaluate the physicochemical and medicinal chemistry characteristics of the tested compounds [37]. Marvin was used for drawing and generating SMILES, which were utilized in ADMETLab 3.0 software, Marvin 17.21.0, Chemaxon (https://www.chemaxon.com).

### 2.16. Molecular Docking

The molecular docking studies of the synthesized compounds were performed utilizing the Schrödinger suit (2024-4 release). The structures of β-Tubulin (PDB: 5LYJ [38]) and PPAPγ (PDB: 4PRG [39]) were obtained from the Protein Data Bank (PDB; http://www.rcsb.org/ accessed on 5 Semptember 2024). The proteins were adjusted by the Protein Preparation Wizard of Schrödinger suit for grid generation and molecular docking. The proteins underwent pre-processing to remove water molecules and ligands from the PDB file. Bond orders were assigned, and pKa values were generated at a pH of 7 ± 2. Further optimization of the proteins was achieved through energy minimization using an OPLS4 force field, resulting in a stable structure suitable for subsequent studies. The Ligprep panel of Schrodinger’s suit was used for building ligands. Optimized potential for liquid simulation (OPLS4) was used for energy minimization. Torsional flexibility was given to obtain a better complementary pose. The grid generation was performed in the area of original co-crystalized ligands of the tested protein structures (Combretastatin A4 for β-Tubulin and 4-[4-[(2S,5S)-5-[2-(dibenzylamino)-2-oxoethyl]-2-heptyl-4-oxo-1,3-thiazolidin-3-yl]butyl]benzoic acid for PPARγ), they also were chosen as reference molecules, respectively. Next, molecular docking was performed using the Glide SP docking protocol in Schrödinger. The obtained results are displayed as docking score and ligand efficiency score [40,41] in kcal/mol. Visualization of the molecular docking results was performed using the Maestro interface of the Schrödinger suit.

### 2.17. Statistical Analysis

To analyze and showcase the obtained results, GraphPad Prism Version 6.0 (San Diego, CA, USA) was utilized. Point to point analysis, one-way and two-way ANOVA tests were applied to obtain the results. The Tukey test was performed for two-way ANOVA tests to adjust for multiple comparisons. Statistically significant difference was defined as * *p* ≤ 0.05; ** *p* ≤ 0.01; *** *p* ≤ 0.001; **** *p* ≤ 0.0001; ns—not statistically significant.

## 3. Results

### 3.1. Synthesis

**Scheme 1:**
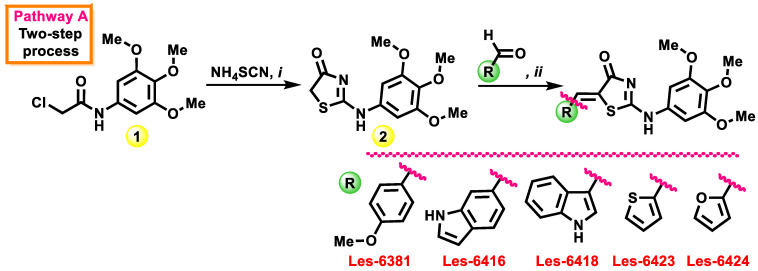
Synthesis of 5-ene-2-((3,4,5-trimethoxyphenyl)amino)thiazol-4(5H)-ones Les-6381, Les-6416, Les-6418, Les-6423, and Les-6424 via two-step approach. Reagents and conditions: **i**—**1** (10 mmol), NH_4_SCN (20 mmol), acetone (10 mL), reflux, 6 h, 64%; **ii—2** (10 mmol), aromatic/heterocyclic aldehyde (12.5 mmol), anhydrous sodium acetate (10 mmol), glacial acetic acid (10 mL), reflux 3–4 h, 58–67%.

Initially, the synthesis of target compounds was performed using a known two-step approach [42] via cyclization of 2-chloro-N-(3,4,5-trimethoxyphenyl)acetamide (**1**) under the action of double excess of ammonium thiocyanate in the acetone medium (Scheme 1). Formed 2-((3,4,5-trimethoxyphenyl)amino)thiazol-4(5H)-one (**2**) was used without any additional purification on the next step in the Knoevenagel condensation with a set of aromatic and heterocyclic aldehydes leading to the corresponding 5-aryl(hetaryl)idene derivatives with good yields (58–67%).

**Scheme 2:**
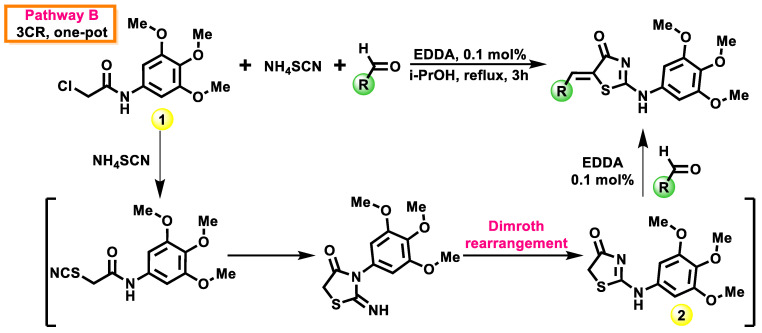
Three-component synthesis of 5-ene-2-((3,4,5-trimethoxyphenyl)amino)thiazol-4(5H)-ones. Reagents and conditions: **i**—**1** (10 mmol), NH_4_SCN (12.5 mmol), aromatic/heterocyclic aldehyde (10 mmol), EDDA (10 µmol), 2-propanol (10 mL), reflux, 3 h, 55–64 %. Such an approach allows for minimizing total reaction time by up to three hours compared with 9–10 h following a two-step protocol. It also provides for the economy of reagents, catalysts, and solvents and allows obtaining the target 5-aryl(hetaryl)idene derivatives with the same yield and purity level.

With an aim to optimize the synthetic process for the abovementioned, the 5-ene-2-((3,4,5-trimethoxyphenyl)amino)thiazol-4(5H)-ones three-component (3CR) protocol was developed and proposed (Scheme 2). The novelty and originality of the reported 3CR process lies in the one-pot interaction of **1**, ammonium thiocyanate, and aromatic/heterocyclic aldehydes with the presence of ethylenediamine diacetate as a catalyst (0.1 mol%) in the 2-propanol medium.

**Scheme 3:**
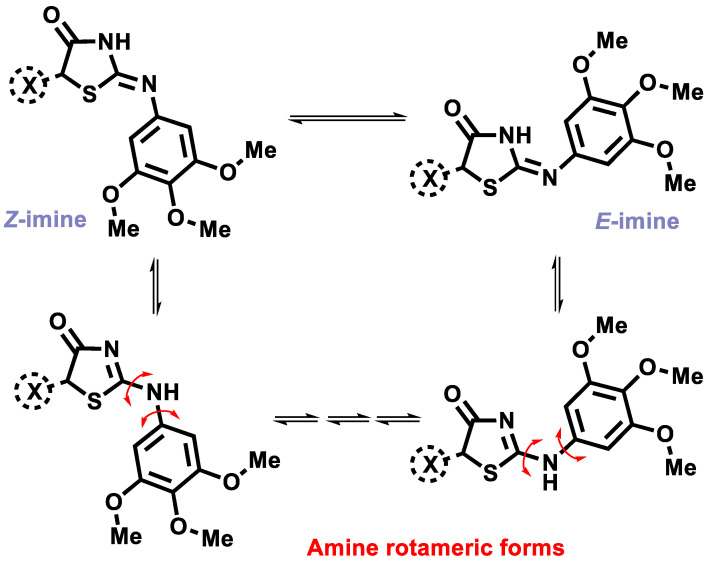
Possible tautomeric/rotameric forms for compounds **2**, Les-6381, Les-6416, Les-6418, Les-6423, and Les-6424.

The structure of synthesized derivatives **2**, Les-6381, Les-6416, Les-6418, Les-6423, and Les-6424 was confirmed using ^1^H, ^13^C, NMR, and LC–MS spectra (copies of spectra are presented in the Supplementary Materials). The spectral pattern of the ^1^H NMR spectra is complicated, and the proton signals are either doublets or triplets due to the presence of prototropic amino/imino tautomerism and rotamerization, as depicted in Scheme 3, and is typical for this 4-thiazolidinones subtype [43,44,45].

### 3.2. In Vitro Anticancer Activity Evaluation in 60 Lines Under the NCI DTP Screening

Synthesized compounds Les-6416 and Les-6418 were tested on a panel of 60 cancer cell lines at one dose assay (10^−5^ M) within the Developmental Therapeutic Program (DTP) by the National Cancer Institute (NCI, Bethesda, MA, USA). Primary anticancer assays (Table 1) were performed according to the NCI protocol, as described before by [28,29,30]. The percentage of growth (GP) was analyzed using a spectrophotometry method versus controls not treated with test agents after 48 h exposure, and using the SRB assay to determine cell viability or growth.

The studied compounds showed pronounced anticancer activity in DTP screening, which is the rationale for their in-depth studies. Thus, Les-6418 inhibited the growth of all 60 tested cancer cell lines by more than 15%, with a range of growth −88.00–14.92%. It is important to note that Les-6418 showed, in most cases, cytotoxic properties, causing the death of cancer cells. Non-small cell lung cancer (NCI-H460 and HOP-92), melanoma MDA-MB-435), ovarian (OVCAR-3), renal (SN12C and A498, 786-0), and CNS (SF-539, U251, and SF-295) cancer cell lines were most sensitive to the action of Les-6418. The range of cell growth under the influence of the Les-6416 was −33.26–97.05% and cytotoxic and significant cytostatic effects were noted for two melanoma lines (MDA-MB-435 and SK-Mel-5), two breast cancer cell lines (MDA-MB-468 and MCF-7) as well as for lung and colon cancer cell lines NCI-H522 and HT29, respectively.

Taking into account the significant inhibition of the viability of tumor cells by Les-6418, it was selected for the in-depth screening of its action towards a full panel of cells in a broad range of concentrations [28,29,30]. The studied derivative inhibited the viability of all tested cancer cell lines at micromolar concentrations (Table 2). The average meaning of the dose–response parameters GI_50_, TGI (molar concentration of the compound leading to the total inhibition), and LC_50_ (molar concentration of the compound leading to 50% net cell death) were 3.27, 18.6, and 56.6 μM, respectively. It is important to note that Les-6418 was active in the submicromolar concentration range of 0.28–0.98 μM (GI_50_) towards the following cell lines: CCRF-CEM, HL-60(TB), SR (leukemia); NCI-H522 (non-small cell lung cancer); HT29, KM12 (colon cancer); MDA-MB-435 (melanoma); OVCAR-3 (ovarian cancer); ACHN (renal cancer); BT-549, MDA-MB-468 (breast cancer). The studied compound showed the highest efficiency against the melanoma line LOX IMVI, because GI_50_, TGI, and LC_50_ parameters were 0.28, 0.94, and 14.2 μM, respectively.

The selectivity indices of the studied Les-6418 were calculated by dividing the full panel MG_MID GI_50_, TGI, and LC_50_ (μM) of the Les-6418 by the individual parameter’s value for each cell line (μM). Ratios that were between three and six meant moderate selectivity; ratios greater than six indicated a high selectivity toward the corresponding cell line, while the compounds not addressing any of these criteria were rated nonselective [46]. At the studied dose–response parameters level, the Les-6418 did not show a selectivity of action against certain types of cancer (SI = 0.71–1.60, Table 2). Minor selectivity was observed at the GI_50_ level for breast cancer (SI = 1.41) and leukemia (SI = 1.60) lines. At the same time, regarding the individual cell lines, the best SI values were observed for the most sensitive line, MDA-MB-435 (melanoma), with SI = 11.8 (GI_50_), 19.8 (TGI), and 3.98 (LC_50_).

### 3.3. Cytotoxic Activity of the Novel Compounds

To investigate the cytotoxic potential of the newly synthesized compounds, a comprehensive assessment was conducted utilizing the MTT assay. This assay was applied on a diverse panel of cell lines, such as human breast cancer cell lines (MCF-7 and MDA-MB-231), human gastric cancer cell line (AGS), human colorectal cancer cell lines (HT-29 and DLD-1), human glioblastoma cancer cell lines (T98G and A172), mouse astrocyte type I clone cells (C8-D1A), primary dermal fibroblast (HDFa), and human normal breast cells (MCF-10A) for 24, 48, and/or 72 h. Half maximal inhibitory concentration (IC_50_) [µM] values were determined using point-to-point analysis and displayed as means ± SD in the table below (Table 3).

**Figure 1:**
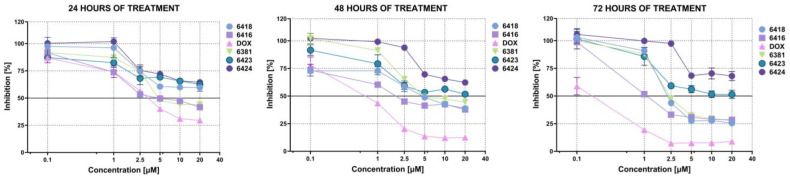
Graphical representation of MCF-7 cells’ viability after treatment with Les-6416, Les-6418, Les-6381, Les-6423, Les-6424, and DOX.

Based on the obtained data, it was determined that newly synthesized compounds showed a potent activity against a number of cell lines, such as MCF-7, DLD-1, HT-29, AGS, A172, and T98G. However, the most notable results were observed against the breast cancer MCF-7 cell line, especially Les-6416, Les-6418, and Les-6381 (Figure 1). While Les-6381 possesses a much broader spectrum of activity against the majority of the tested cell lines, it also displayed a higher level of harmful effect on normal cells, particularly against MCF-10A-human normal epithelial breast cells. In comparison, the other two active compounds, Les-6416 and Les-6418, displayed a higher degree of selective activity, mainly against MCF-7, AGS, and HT-29 cells. As a result of those observations, it was determined that Les-6381 does not possess selectivity and its damaging effects on normal cells are greater than its cytotoxic activity. Les-6416 and Les-6418 possessed a lesser degree of negative impact on normal cells, including MCF-10A, C8-D1A, and primary dermal fibroblast (HDFa). For those reasons, the MCF-7 cell line and Les-6416 and Les-6418 were chosen for future studies.

### 3.4. Antiproliferative Activity Effect of the 4-Thiazolidinone Derivatives on Human Breast Cancer Cells

**Figure 2:**
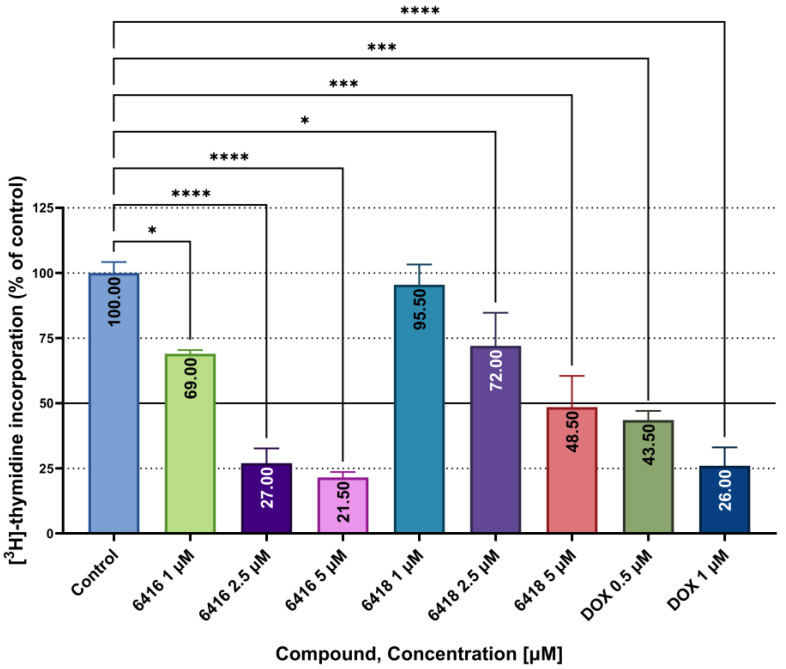
Incorporation of [^3^H]-thymidine into MCF-7 cells after 24 h of treatment with Les-6416, Les-6418 (5, 2.5, and 1 µM), and DOX (1, 0.5 µM). The results are shown as means ± SD. To demonstrate the difference between the obtained results in comparison to the control, one-way ANOVA analysis was utilized. **** *p* ≤ 0.0001; *** *p* ≤ 0.001; * *p* ≤ 0.05.

To investigate the antiproliferative activity of Les-6416 and Les-6418 on the MCF-7 cell line after 24 h of treatment, the [^3^H]-thymidine incorporation assay was applied. Based on the outcome of previous analysis and the structural similarity of the tested compounds, 5, 2.5, and 1 µM concentrations were applied for this research. The obtained results are shown in the graph below (Figure 2). Interestingly, Les-6416, despite having a substantial structural similarity to Les-6418, displayed a more significant antiproliferative activity, with 21.50 ± 2.12% at concentration of 5 µM, 27.00 ± 5.66% at 2.5 µM and 69 ± 1.41% of [^3^H]-thymidine incorporation relative to the control at 1µM, while Les-6418 possessed only 48.50 ± 12.02%, 72.00 ± 12.73% and 95.50 ± 7.78% for 5, 2.5, and 1 µM accordingly. Formidable results also were obtained from the reference drug doxorubicin hydrochloride: 26.00 ± 7.07% for 1 µM and 43.50 ± 3.54% for 0.5 µM

### 3.5. Clonogenic Assay 

**Figure 3:**
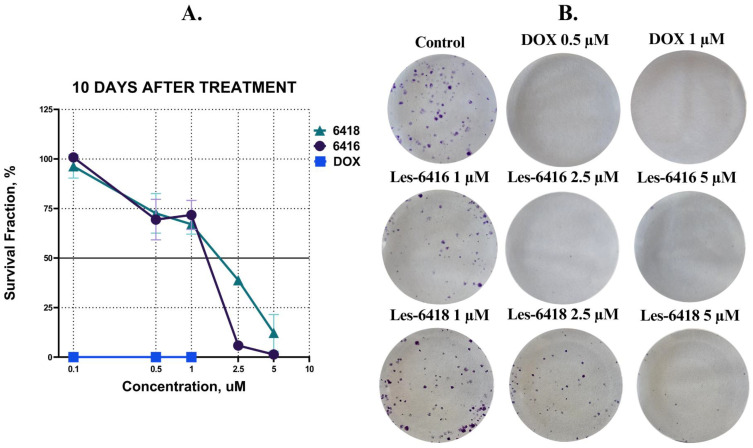
(**A**) Graphical representation of MCF-7 cell survival fraction following a 24 h treatment period, assessed at the 10-day endpoint. (**B**) Representative pictures of cell culture wells of MCF-7 cell survival fraction following a 24 h treatment period, assessed at the 10-day endpoint.

To further demonstrate the long-term effects of the tested compounds on MCF-7 cells’ viability after 24 h of treatment, we applied a clonogenic assay. Colonies formation was assessed after a 10-day incubation period following the treatment. While both of the tested compounds showed an inhibitory effect on the formation of colonies, Les-6416 displayed more potent activity, with 1.35 ± 1.42% for the concentration of 5 µM and 5.89 ± 0.71% survival fraction for 2.5 µM. At the same time, Les-6418 had only 12.27 ± 9.27% and 38.83 ± 1.66% of survival fraction for 5 and 2.5 µM, accordingly. Interestingly enough, concentrations 1, 0.5, and 0.1 µM had similarly mediocre effects for both compounds. Reference drug doxorubicin hydrochloride displayed a 0% survival fraction in all 3 concentrations: 1, 0.5, and 0.1 µM (Figure 3).

### 3.6. Effects of the New 4-Thiazolidinone Derivatives on Cell Migration

**Figure 4:**
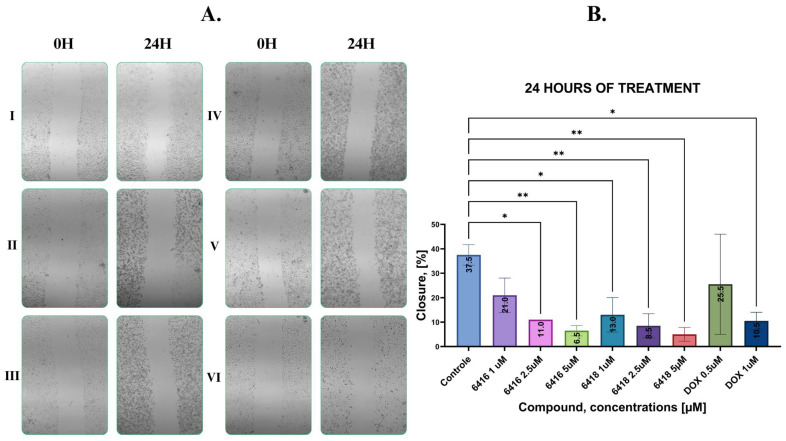
(**A**) Representative images of the MCF-7 cell monolayers before and after 24 h treatment: I—control, II—Les-6416 5 µM, III—Les-6416 2.5 µM, IV—Les-6418 5 µM, V—Les-6418 2.5 µM, and VI—DOX 1 µM; (**B**) graphical representation of the obtained and analyzed results. A one-way ANOVA test was applied to determine statistical significance. * *p* ≤ 0.05, ** *p* ≤ 0.01.

To examine the impact of Les-6416 and Les-6418 on cell migration, the scratch assay was performed. The MCF-7 breast cancer cells were treated for 24 h with the tested compounds in concentrations of 5, 2.5, and 1 µM and doxorubicin hydrochloride in concentrations of 1 and 0.5 µM. The obtained results are shown in Figure 4. All of the tested compounds and concentrations displayed a statistically significant result in comparison to untreated cells (control) with 37.50 ± 4.20% of reduction in the gap area, except cells treated with 1 µM of Les-6416 and 0.5 µM of DOX, which had 21.00 ± 7.07% and 25.50 ± 20.51% closure of the gap area, respectively. The obtained results displayed that the majority of the compounds and concentrations did not allow the cells to migrate into the wound, as such displaying a potent inhibitory effect on cell migration.

### 3.7. Proapoptotic Activity of the Synthesized Compounds

**Figure 5:**
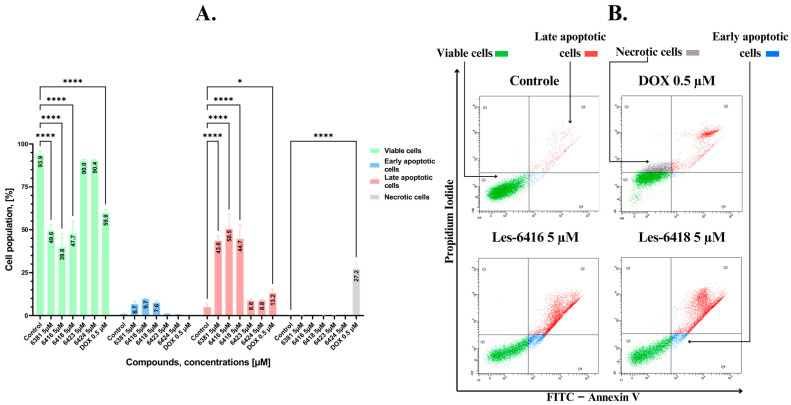
Flow cytometry assessment of MCF-7 breast cancer cells after 24 h of incubation with tested compounds (Les-6416, Les-6418, Les-6381, Les-6423, Les-6424) in a concentration of 5 µM and reference drug (doxorubicin hydrochloride) in a concentration of 0.5 µM. (**A**) Graph showing the obtained results in comparison to the control (untreated cells). A two-way ANOVA test was applied to determine statistical significance. The Tukey test was utilized to adjust for multiple comparisons. * *p* ≤ 0.05; **** *p* ≤ 0.0001. (**B**) Representative cytograms obtained from flow cytometry analysis.

To assess the effects of novel compounds on apoptosis, we utilized flow cytometry analysis with Annexin V-FITC and Propidium Iodide double staining. In this way, we could distinguish between different cell populations based on their state: unstained or alive cells, cells stained with propidium iodide or necrotic cells, cells only stained with FITC-labeled annexin V or early apoptotic cells, and cells stained with both annexin V and propidium iodide or late apoptotic cells. As a result of 24 h of treatment on MCF-7 cells in 5 µM concentration for novel compounds and 0.5 µM for reference drug, we observed that Les-6416, Les-6418, and Les-6381 proved to be the most potent, while Les-6423 and Les-6424 did not have any statistically significant results in comparison to the control. With 8.23 ± 2.56% early apoptotic cells, 45.63 ± 10.29% late apoptotic cells and 0.07 ± 0.06% necrotic cells Les-6416 exhibited the most significant effect as a proapoptotic compound, followed by Les-6418 with 7.55 ± 1.17%, 44.68 ± 8.18%, and 0.13 ± 0.05% of early apoptotic, late apoptotic, and necrotic cells, respectively. The only compound that showed a significant increase in necrotic cells was the reference drug doxorubicin hydrochloride, with 27.23 ± 3.76% (Figure 5). 

### 3.8. Activation of Caspases 7, 8, 9, 10 in Human Breast Cancer Cells

**Figure 6:**
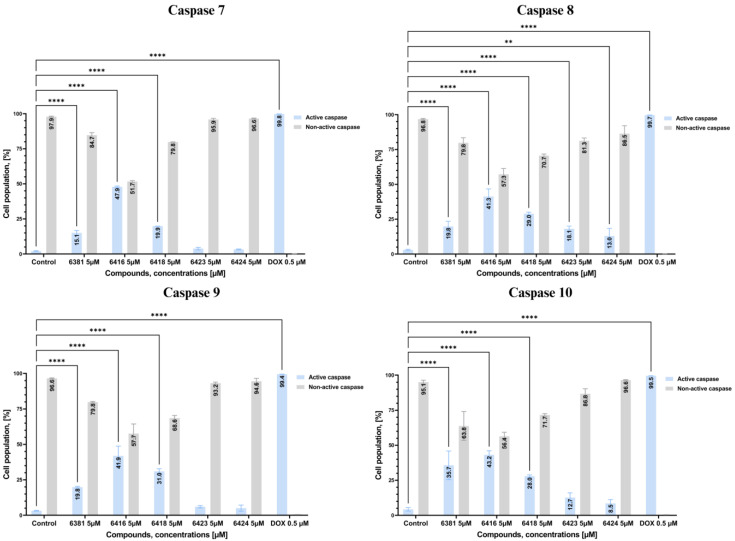
Graphs showing the results of flow cytometry measurement of caspases 3, 8, 9, and 10 activities in MCF-7 cells after 24 h incubation with tested compounds (Les-6416, Les-6418, Les-6381, Les-6423, and Les-6424) in concentration of 5 µM and reference drug (doxorubicin hydrochloride) in concentration of 0.5 µM in comparison to the control (untreated cells). A two-way ANOVA test was applied to determine statistical significance. The Tukey test was utilized to adjust for multiple comparisons. **** *p* ≤ 0.0001; ** *p* ≤ 0.01; ns—not statistically significant.

To further investigate the effects of the newly synthesized compounds on the process of apoptosis we decided to measure their impact on various caspases as evidence of intrinsic (caspase 9) or extrinsic (caspase 8, 10) pathways of programmed cell death by applying flow cytometry analysis after 24 h treatment of MCF-7 breast cancer cells with novel 4-thiazolidinone derivatives and reference drug doxorubicin hydrochloride. Compounds that showed a proapoptotic effect in the previous tests, mainly Les-6416, Les-6418, and Les-6381, also displayed a potent impact on all caspases. With 41.28 ± 5.45%, 43.23 ± 2.85%, and 41.90 ± 6.89% of cells with activated caspase 8, 10, and 9, respectively, Les-6416 continued the trend of displaying the most significant effect on the three tested caspases. Les-6418 and Les-6381 also showed a statistically significant impact on caspases 8, 9, and 10. Interestingly enough, Les-6423 was comparable to Les-6381 regarding the effect on caspase 8, with 18.13 ± 1.96% and 19.8 ± 3.69% of cells with activated caspase 8, respectively. However, Les-6423 did not show any effect on caspases 9 or 10. Both the intrinsic and extrinsic apoptotic pathways converge into a unified pathway, initiating the execution phase of apoptosis. During the course of this phase, executioner caspases 3 and 7 were formed. However, MCF-7 cells do not express caspase 3; as such, only caspase 7 was formed [47]. The obtained results showed that among newly synthesized 4-thiazolidinones, only Les-6416, Les-6418, and Les-6381 possessed a statistically significant effect on caspase 7 activation with 47.90 ± 0.57%, 19.85 ± 0.07%, and 15.05 ± 1.73% of cells with activated caspase, respectively (Figure 6).

### 3.9. Effects of Novel Compounds on Mitochondrial Membrane Potential

**Figure 7:**
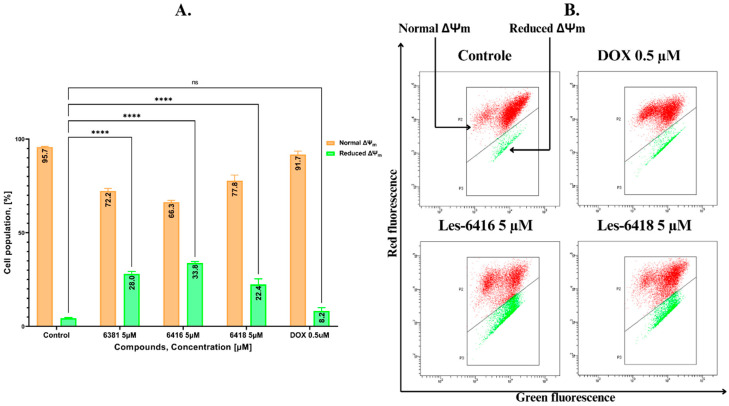
Flow cytometry assessment of differences in the mitochondrial membrane potential of MCF-7 breast cancer cells after 24 h of incubation with tested compounds (Les-6416, Les-6418, and Les-6381) in a concentration of 5 µM and reference drug (doxorubicin hydrochloride) in a concentration of 0.5 µM. (**A**) Graph showing the obtained results in comparison to the control (untreated cells). A two-way ANOVA test was applied to determine statistical significance. The Tukey test was utilized to adjust for multiple comparisons. **** *p* ≤ 0.0001; ns—not statistically significant. (**B**) Representative pictures of cytograms from the flow cytometry analysis.

A critical determinant of the induction of the intrinsic pathway of apoptosis is the decrease in mitochondrial membrane potential (ΔΨm). To investigate the effects of the novel 4-thiazolidinones on mitochondrial membrane potential, flow cytometry analysis of JC-1 dye was applied. JC-1 is a carbocyanine lipophilic cationic fluorochrome whose fluorescence depends on the MMP. Red fluorescence is emitted in viable cells with healthy mitochondria; however, apoptotic or necrotic cells with damaged mitochondria emit green fluorescence. MCF-7 cells were treated with the most potent synthesized compounds for 24 h. The obtained results displayed that those compounds decreased the potential of mitochondrial membrane for 33.80 ± 0.85%, 22.35 ± 3.06%, and 27.95 ± 1.43% for Les-6416, Les-6418, and Les-6381, respectively, further confirming the effects of the tested compounds on inducing the intrinsic pathway of apoptosis (Figure 7).

### 3.10. Increase in p53 and Cytochrome C Concentration in Human Breast Cancer Cells After Treatment with Novel 4-Thiazolidinones Derivatives

**Figure 8:**
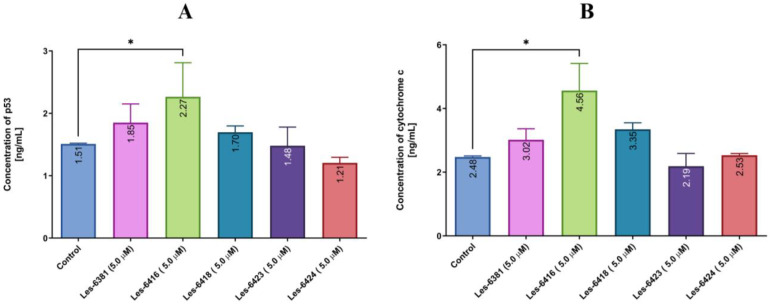
The concentration of p53 (**A**) and cytochrome C (**B**) protein in MCF-7 breast cancer cells after 24 h incubation with the tested compounds (5 µM). The one-way ANOVA test was utilized to demonstrate the comparison of the tested compounds to the control. * *p* ≤ 0.05.

Both p53 and cytochrome C proteins play pivotal roles in the process of apoptosis. For this reason, concentrations of the mentioned proteins were measured in MCF-7 cells after 24 h treatment with the tested compounds (Les-6416, Les-6418, Les-6381, Les-6423, Les-6424) in concentrations of 5 µM. From the obtained results, it was noted that while Les-6418 and Les-6381 displayed a slight increase in concentration of both proteins, only Les-6416, as the most potent compound, had a statistically significant result with 2.27 ± 0.55 and 4.56 ± 0.85 ng/mL of p53 and cytochrome C concentration accordingly (Figure 8).

### 3.11. Increase in Bax Positive Cell Population in Human Breast Cancer Cells After Treatment with Novel 4-Thiazolidinones Derivatives

**Figure 9:**
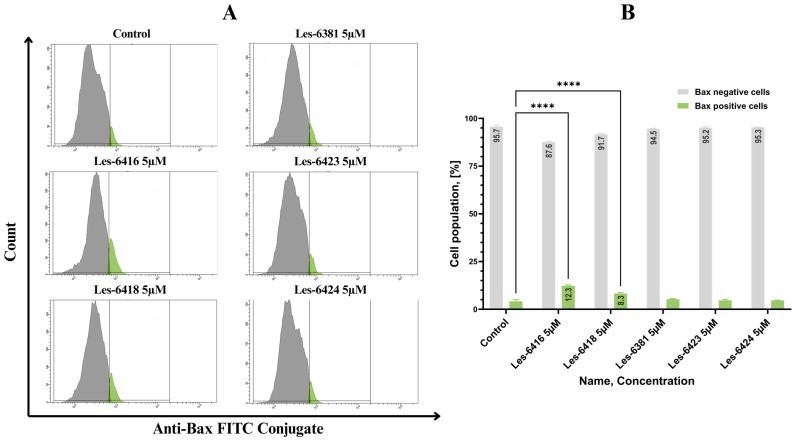
Flow cytometric analysis of MCF-7 cells after 24 h of treatment by novel 4-thiazolidinone derivatives (Les-6416, Les-6418, Les-6381, Les-6423, Les-6424) in a concentration equal to 5 µM in comparison to the untreated cells (control) with anti-Bax antibody. (**A**) Representative cytograms obtained from flow cytometry analysis. (**B**) Graph showing the obtained results in comparison to the control (untreated cells). A two-way ANOVA test was applied to determine statistical significance. The Tukey test was utilized to adjust for multiple comparisons. **** *p* ≤ 0.0001.

Another important protein for the intrinsic pathway of apoptosis is a pro-apoptotic member of the Bcl-2 family-Bax. To analyze the effects of novel compounds on this protein, flow cytometric analysis of MCF-7 cells after 24 h of treatment by the tested structure (Les-6416, Les-6418, Les-6381, Les-6423, Les-6424) in a concentration equal to 5 µM was performed. The obtained results showed that the lead compound (Les-6416) displayed the most potent effect on Bax induction, with 12.27 ± 0.45% of Bax-positive cells. The only other compound among the tested that displayed a statistically significant result is Les-6418, with 8.27 ± 0.58% of Bax-positive cells (Figure 9).

### 3.12. Increase In ROS Generation

**Figure 10:**
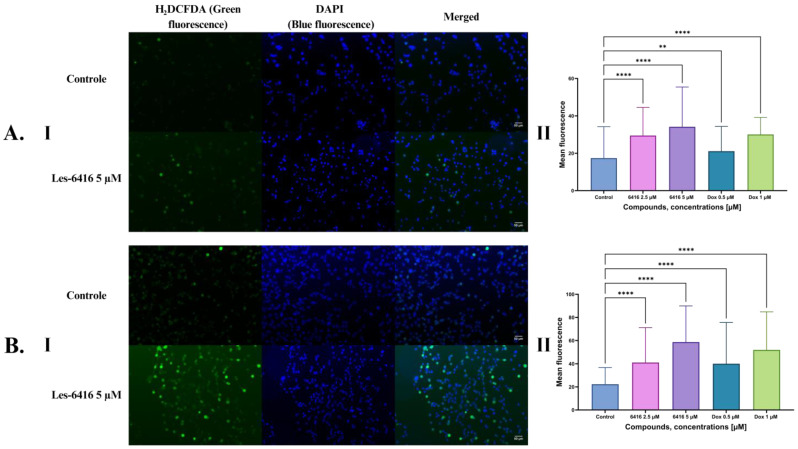
Results of H_2_DCFDA and DAPI double staining on MCF-7 cells. (**A**) After 1 h treatment: I—representative microscope images, II—graph showing the obtained results with one-way ANOVA test. (**B**) After 3 h treatment: I—representative microscope images, II—graph showing the obtained results with one-way ANOVA test. N > 300, **** *p* ≤ 0.0001; ** *p* ≤ 0.01.

To evaluate the influence of the most efficacious compound, Les-6416, and the reference drug on the generation of reactive oxygen species, a double staining method was applied. H_2_DCFDA (green fluorescence) was utilized to mark oxidative stress, and DAPI (blue fluorescence) to stain nuclear genetic material. Les-6416 induced a statistically significant increase in oxidative stress in both concentrations (5 µM, 2.5 µM) after 1 h of treatment and especially so after 3 h of treatment. Conversely, DOX in a concentration of 0.5 µM after one hour of treatment had the least consequential effect on oxidative stress. However, this changed after three hours of treatment, where DOX in the concentration of 0.5 µM showcased a comparable result to Les-6416 in the concentration of 2.5 µM, which was approximately two times more than the untreated control. The most impressive effect on stress generation was noticed after treatment with Les-6416 in the concentration of 5 µM, which was comparatively two times more than the control after 1 h and approximately a threefold increase in comparison to the control after 3 h of treatment. These observations displayed the time- and activity-dependent effects of Les-6416 on the generation of reactive oxygen species in breast cancer cells (Figure 10).

### 3.13. Morphological Analysis of Cells by Acridine Orange and Ethidium Bromide Double Staining

**Figure 11:**
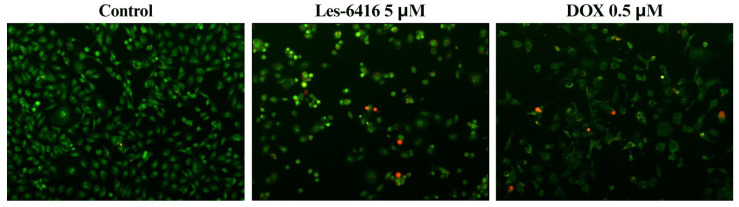
Representative microscope images of acridine orange/ethidium bromide stained MCF-7 cells after 24 h of treatment by lead compound–Les-6416 in concentration equal to 5 µM and DOX in concentration equal to 0.5 µM as a positive control. Green indicates living cells, while red indicates dead cells.

To analyze morphological changes in MCF-7 cells after 24 h of treatment, we performed the double staining with acridine orange and ethidium bromide. This method provides the capability to observe different stages of cell death depending on cell membrane integrity and chromatin condensation. Green fluorescence can be observed due to acridine orange staining, that penetrates all cells, while red coloring is contributed to ethidium bromide, which can only penetrate cells with injured cell membranes. Representative images on the figure below (Figure 11) showcase a visible reduction in cell population, when treated by lead compound–Les-6416. Treated cells also displayed noticeable morphological changes such as cell shrinkage and chromatin condensation, which are known signs of the programmed cell death.

### 3.14. In Silico ADMET Evaluation and Molecular Docking

**Figure 12:**
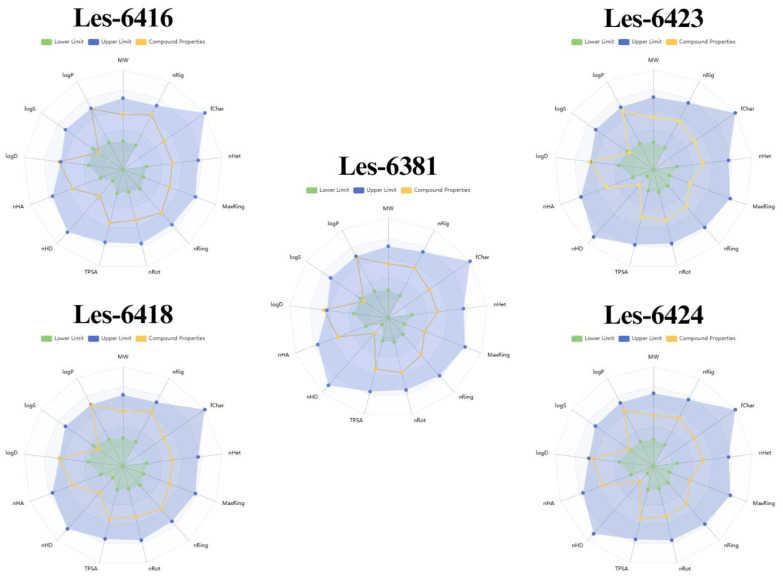
Bioradars of the newly synthesized compounds, predicted and generated by the ADMETLab 3.0 tool. MW—molecular weight; nRig—number of rigid bonds; fChar—formal charge; nHet—number of heteroatoms; MaxRing—number of atoms in the biggest ring; nRing—number of rings; nRot—number of rotatable bonds; TPSA—Topological polar surface area; nHD—number of hydrogen bond donors; nHA—number of hydrogen bond acceptors; logD—logarithm of the n-octanol/water distribution coefficients at pH = 7.4; logS—logarithm of aqueous solubility value; logP—logarithm of the n-octanol/water distribution coefficient.

For a compound to be a viable drug candidate, cytotoxic activity alone is insufficient. Consequently, a suite of rules and characteristics has been developed to evaluate a compound’s absorption, distribution, metabolism, excretion, and toxicity (ADMET) profiles. With the advancement of technology, those characteristics can be tested using an in silico approach. As such, ADMET evaluation of the tested compounds was performed by utilizing the ADMETLab 3.0 web application with pre-prepared SMILES strings, generated by Marvin software. The bioavailability radars (bioradars) of the novel 4-thiazolidinones can be seen in Figure 12. The compounds did not break through the upper limit of all characteristics, except logD. Les-6416, Les-6418, Les-6381, and Les-6423 had slightly higher logD than the upper limit. Furthermore, none of the tested compounds broke the Lipinski and Pfizer rules, and all of them also satisfied the Golden Triangle rule. Interestingly enough, the bioavailability of the tested compounds significantly differs. Les-6416 showed the most promising results for 20% and 30% bioavailability. Despite being incredibly structurally similar, Les-6418 had a notably lesser result for F_30%_ (Table 4). This can be one of the potential reasons for the difference in activity of those two compounds. While the obtained results are promising, they will require further validation in vitro, as various factors, such as dose and time exposure, are not included when performing in silico analysis.

**Figure 13:**
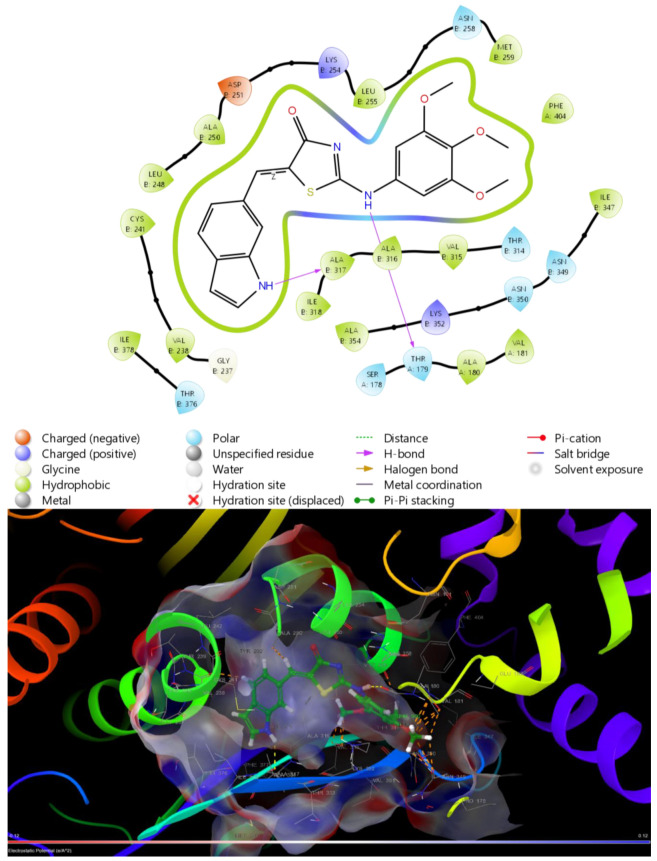
Binding site and ligand interactions for Les-6416 obtained from molecular docking with β-Tubulin (5LYJ).

Another popular in silico research method is molecular docking. It allows us to explore various possible molecular targets for the tested compounds and their potential to affect said targets. β-Tubulin (PDB: 5LYJ [38]) and PPAPγ (PDB: 4RPG [39]) were chosen as possible molecular targets due to the previous 4-thiazolidinone derivatives with confirmed effects on them [11,44]. Novel compounds showed a potent affinity towards β-Tubulin, with Les-6416 and Les-6416 having more prominent binding energy than even the original ligand of the tested target, Combretastatin A4 (Table 5, Figure 13). The obtained results displayed a path for future research regarding synthesized compounds.

## 4. Discussion

4-Thiazolidinone derivatives with anticancer activity have been previously reported [7,52,53,54,55]. Consequently, it was not surprising that synthesized structures exhibited a potent effect on inhibiting cell metabolism across various cell lines, with MCF-7 cells being the most sensitive to the effects of the novel compounds, Les-6416, Les-6418, and Les-6381 in particular. While only Les-6416 displayed an IC_50_ equal to 4.70 ± 0.06 µM after 24 h of treatment, it was decided in considering their structural similarity, that compounds would be tested mainly at a concentration of 5 µM on this cell line. Compounds Les-6423, Les-6424, and Les-6381 were excluded from some of the performed tests due to their inadequate activity (Les-6423, Les-6424) or potent toxic effects on normal human cell lines (Les-6381).

Antiproliferative effects of Les-6416 and Les-6418 were investigated utilizing [^3^H]-thymidine incorporation and clonogenic assays. Both compounds demonstrated a significant capacity to inhibit MCF-7 cells proliferation at a concentration of 5 µM after 24 h of treatment. Intriguingly, newly synthesized compounds showed a greater inhibitory effect on cell proliferation in comparison to cell metabolism.

Cell migration, a critical process in various biological phenomena, including embryogenesis, tissue remodeling, immune responses, and cancer metastasis [56]. To investigate the effects of the novel compounds, primarily Les-6416 and Les-6418, on MCF-7 cell migration, a scratch was conducted. After 24 h of treatment, both compounds exhibited a profound inhibitory effect on cell migration at a concentration of 5 µM.

To gain a more comprehensive understanding of the anticancer activity of the tested structures, we decided to evaluate their ability to trigger apoptosis. This process is often deregulated in various types of cancer, leading to uncontrolled tumor growth and resistance against a number of existing chemotherapeutics. By targeting apoptosis, the compounds activity will be more focused on affecting cancerous cells that rely on the deregulated process of programmed cell death, as a result, the selectivity of the synthesized compounds should increase [57,58]. The tested compounds showed a significant effect on apoptosis induction, where Les-6416 achieved 8.23 ± 2.56% early apoptotic cells and 45.63 ± 10.29% late apoptotic cells with only 0.07 ± 0.06% necrotic cells. At the same time, Les-6418 also displayed a potent proapoptotic effect with 7.55 ± 1.17%, 44.68 ± 8.18%, and 0.13 ± 0.05% of early apoptotic, late apoptotic, and necrotic cells, respectively. To further characterize the apoptotic mechanisms, we explored the compounds impact on the two primary apoptotic pathways: intrinsic (mitochondria-mediated) and extrinsic (death receptor-mediated). To confirm the effects of novel 4-thiazolidinone derivatives on the death-receptor mediated pathway, we explored the compounds’ effects on activation of caspase 8 and 10, considering their importance in this process [59]. Les-6416 and Les-6418 continued the trend, showing significant effects on the activation of those two caspases. We explored the effects of these compounds on the mitochondria-mediated pathway as well. Their effects on the activation of caspase 9 and reducing mitochondrial membrane potential were investigated, as those are important markers of the intrinsic pathway [59]. The tested compounds, mainly Les-6416, Les-6418, and Les-6381, displayed promising results, suggesting that in addition to affecting the extrinsic pathway of apoptosis, the novel structures also induce the intrinsic pathway correspondingly.

To further expand our investigation of the effects of synthesized compounds on apoptotic mechanisms, we examined their influence on p53, cytochrome C, and Bax proteins. The p53 protein is known to have an inducing effect on both pathways of programmed cell death [60], p. 53. Cytochrome C and Bax, on the other hand, are renowned markers of the intrinsic pathway of apoptosis [61]. After performing ELISA tests to determine the effect of the tested compounds on p53 and Cytochrome C, we found that Les-6416 possessed the most significant effect on them. The flow cytometric assessment of Bax protein in treated cells further confirmed this trend. Similar results were reported for FDA-approved 4-thiazolidinone derivatives. Rosiglitazone, for example, displayed the capability to increase p53 and caspase 9 protein levels in MCF-7 cancer cells [62].

**Figure 14:**
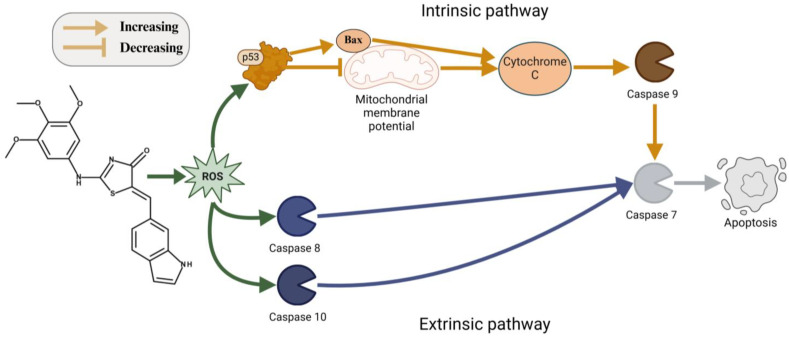
ROS-mediated mechanism of apoptosis induction of Les-6416.

4-Thiazolidinone derivatives are characterized by increasing ROS generation in cancer cells [26]. This may lead to inducing the process of apoptosis by either intrinsic or extrinsic pathways. The mitochondrial pathway can be induced by reactive oxygen species through activation of p53 [63]. This will activate the pro-apoptotic Bcl-2 family of proteins. ROS can also cause the oxidation of cardiolipin, resulting in the release of cytochrome C into the cytosol. Furthermore, under the effects of the ROS mitochondrial membrane, depolarization can occur, opening the Bax/Bak channels, leading to the release of cytochrome C to the cytosol. Afterwards, cytochrome C will form an apoptosome in the cytosol together with procaspase-9 and Apaf-1, activating caspase 9. This will instigate the activation of effector caspases -3, 6, and 7, resulting in a programmed cell death [63]. The extrinsic pathway can also be induced by ROS generation through activation of death receptors such as Fas, TRAIL-R_1/2,_ and TNF-R_1_. This will initiate the FADD protein and procaspases 8 and 10, which will lead to the activation of caspases 8 and 10, respectively. Those caspases will activate the effector caspases -3, 6, and 7, initiating cell death via apoptosis [63]. Since Les-6416 displayed a significant effect on p53, cytochrome C, and Bax proteins, we decided to test this compound capability to increase ROS generation in MCF-7 cells. Les-6416 showed a threefold increase in comparison to the control in the concentration of 5 µM after 3 h of treatment. Those results suggest that one of the mechanisms of anticancer activity for Les-6416 is through increasing ROS generation in cancer cells (Figure 14). The obtained results correlate with the literature information regarding the effects of reactive oxygen species generation on anticancer activity. For instance, frequently utilized drugs, such as cisplatin and doxorubicin, are known to directly induce ROS generation and, through that, to promote the process of programmed cell death, which is confirmed by increased expressions of numerous apoptosis-related proteins: p53, Bax, caspase 3, TNF, and FAS [64].

## 5. Conclusions

A series of 5-ene-2-((3,4,5-trimethoxyphenyl)amino)thiazol-4(5*H*)-ones were designed and synthesized as potential anticancer agents. An effective multicomponent synthetic protocol based on one-pot interaction of 2-chloro-*N*-(3,4,5-trimethoxyphenyl)acetamide, ammonium thiocyanate, and aromatic/heterocyclic aldehydes with ethylenediamine diacetate catalyst in the 2-propanol medium was developed for the obtaining of target derivatives. The proposed protocol could be used as a general approach for the synthesis of 5-ene-2-aminothiazol-4(5*H*)-ones.

The studies conducted in vitro revealed that novel 4-thiazolidinone derivatives Les-6416, Les-6418, and Les-6381 exhibited a significant ability to inhibit cell proliferation and cell metabolism of breast cancer MCF-7 cells. Notably, Les-6416 and Les-6418 demonstrated lower toxicity towards normal cells, primary dermal fibroblast (HDFa), MCF-10A (human normal epithelial breast cells), and C8-D1A (mouse astrocyte type I clone). In contrast, Les-6381 exhibited a higher cytotoxic impact on normal human cells. Further investigations into the anticancer activity of the novel compounds revealed that Les-6416, Les-6418, and Les-6381 displayed the ability to induce apoptosis and its extrinsic and intrinsic pathways by activating caspases 7, 8, 9, and 10 and reducing the mitochondrial membrane potential in MCF-7 breast cancer cells after 24 h of treatment. Nonetheless, Les-6416 showed the most significant effects on crucial pro-apoptotic proteins such as p53, Cytochrome C, and Bax. Additionally, this compound also significantly increased reactive oxygen species generation after 1 and 3 h of treatment, indicating that one of the potential mechanisms of its anticancer effects is through the generation of ROS, which will trigger the activation of both pathways of programmed cell death. Considering the obtained data, novel 4-thiazolidinone derivatives displayed a potential to become new anticancer agents, and further in-depth analysis of the compound’s pharmacokinetics and toxicity profiles should be conducted in future research.

## Figures and Tables

**Table 1:** Anticancer screening data in concentration 10 μM.

Comp.	Mean Growth, %	Range of Growth, %	Top 10 Most Sensitive Cell Lines/Growth %	Positive Cytostatic Effect ^a^	Positive Cytotoxic Effect ^b^
Les-6416	39.56	−33.26–97.05	MDA-MB-435 (Melanoma)/−33.26 SK-Mel-5 (Melanoma)/−4.74 MDA-MB-468 (Breast Cancer)/1.33 NCI-H522 (Non-small Cell Lung Cancer)/3.77 SF-295 (CNS Cancer)/5.01 MCF-7 (Breast Cancer)/6.76 HT29 (Colon Cancer)/7.66 HL60(TB) (Leukemia)/15.10 NCI-H460 (Non-small Cell Lung Cancer)/17.93 UACC-62 (Melanoma)/18.03	38/58	2/58
Les-6418	−43.64	−88.00–14.92	SN12C (Renal Cancer)/−88.00 SF-539 (CNS Cancer)/−85.30 U-251 (CNS Cancer)/−79.79 NCI-H460 (Non-small Cell Lung Cancer)/−77.59 MDA-MB-435 (Melanoma)/−77.11 OVCAR-3 (Ovarian Cancer)/76.96 A498 (Renal Cancer)/−76.12 SF-295 (CNS Cancer)/−73.38 786-0 (Renal Cancer)/−73.06 HOP-92 (Non-small Cell Lung Cancer)/−72.26	7/59	52/59

^a^ Ratio between number of cell lines with GP from 0 to 50 and total number of cell lines. ^b^ Ratio between number of cell lines with GP of <0 and total number of cell lines.

**Table 2:** Influence of Les-6418 on the growth of individual tumor cell lines.

Cell Line	GI_50_, μM/SI	TGI, μM/SI	LC_50_, μM/SI
**Leukemia**			
CCRF-CEM	2.09/1.56	>100/0.19	>100/0.57
HL-60(TB)	0.855/3.82	11.1/1.68	>100/0.57
K-562	0.526/6.22	31.1/0.60	>100/0.57
MOLT-4	2.63/1.24	26.0/0.72	>100/57
RPMI-8226	5.73/0.57	>100/0.19	>100/0.57
SR	0.394/8.30	29.9/0.62	>100/0.57
**MG_MID**	**2.04/1.60**	**49.7/0.37**	**>100/0.57**
**Non-small Cell Lung Cancer**			
A549/ATCC	2.84/1.15	16.5/1.13	73.6/0.77
EKVX	8.02/0.40	22.2/0.84	53.2/1.06
HOP-62	2.69/1.22	15.7/1.18	65.9/0.86
HOP-92	1.98/1.65	6.38/2.92	42.0/1.35
NCI-H266	3.18/1.03	17.4/1.07	68.8/0.82
NCI-H23	5.33/0.61	21.1/0.88	54.0/1.05
NCI-H322M	6.38/0.51	21.2/0.88	54.2/1.04
NCI-H460	2.10/1.56	14.3/1.30	92.0/0.61
NCI-H522	0.403/8.11	5.56/3.34	46.0/1.23
**MG_MID**	**3.66/0.89**	**15.6/1.19**	**61.1/0.92**
**Colon Cancer**			
COLO 205	3.14/1.04	16.4/1.13	49.6/1.14
HCC -2998	6.36/0.51	20.7/0.90	47.8/1.18
HCT-116	1.10/2.97	10.2/1.82	50.6/1.12
HCT-15	1.79/1.83	12.4/1.50	37.7/1.50
HT29	0.923/3.54	10.1/1.84	47.6/1.19
KM12	0.977/3.35	16.8/1.11	70.7/0.80
SW-620	3.10/1.05	21.8/0.85	85.0/0.66
**MG_MID**	**2.48/1.32**	**15.5/1.20**	**55.6/1.02**
**CNS Cancer**			
SF-268	3.56/0.92	20.0/0.93	81.3/0.69
SF-295	2.27/1.44	13.4/1.39	40.9/1.38
SF-539	2.49/1.31	9.95/1.87	35.5/1.59
SNB-19	6.60/0.50	21.2/0.88	52.2/1.08
SNB-75	1.24/2.64	3.63/5.12	11.9/4.75
U251	3.27/1.00	15.7/1.18	54.7/1.03
**MG_MID**	**3.24/1.01**	**14.0/1.33**	**46.1/1.23**
**Melanoma**			
LOX IMVI	2.82/1.16	13.9/1.34	40.9/1.38
MALME -3M	2.01/1.63	15.2/1.22	43.9/1.29
M14	2.02/1.62	15.9/1.17	55.6/1.02
MDA-MB-435	0.280/11.7	0.940/19.8	14.2/3.98
SK-MEL-2	1.45/2.26	12.2/1.52	43.1/1.31
SK-MEL-28	6.07/0.54	21.0/0.89	49.9/1.13
SK-MEL-5	2.46/1.33	14.1/1.32	39.1/1.45
UACC-257	5.18/0.63	21.1/0.88	52.7/1.07
UACC-62	4.75/0.69	19.7/0.94	48.3/1.17
**MG_MID**	**3.00/1.09**	**14.9/1.25**	**43.1/1.31**
**Ovarian Cancer**			
IGROV1	4.91/0.67	21.6/0.86	57.2/0.99
OVCAR-3	0.517/6.32	5.32/3.50	34.0/1.66
OVCAR-4	2.88/1.13	10.6/1.75	35.3/1.60
OVCAR-5	4.58/0.71	17.7/1.05	44.1/1.28
OVCAR-8	10.6/0.31	27.2/0.68	70.1/0.81
NCI/ADR-RES	6.24/0.52	24.2/0.77	74.2/0.76
SK-OV-3	2.56/1.28	10.3/1.81	38.5/1.47
**MG_MID**	**4.61/0.71**	**16.7/1.11**	**50.5/1.12**
**Renal Cancer**			
786-0	2.72/1.20	11.9/1.56	55.7/1.01
A498	10.2/0.32	24.6/0.76	59.6/0.95
ACHN	3.20/1.02	12.0/1.55	36.1/1.57
CAKI-1	0.840/3.89	10.3/1.81	33.1/1.71
RXF 393	1.00/3.27	5.80/3.21	34.0/1.66
SN12C	3.85/0.85	15.1/1.23	43.6/1.30
TK-10	8.56/0.38	22.8/0.82	55.6/1.02
UO-31	3.43/0.95	13.7/1.36	38.8/1.46
**MG_MID**	**4.22/0.77**	**14.5/1.28**	**44.6/1.27**
**Prostate Cancer**			
PC-3	2.59/1.26	22.6/0.82	94.2/0.60
DU-145	4.63/0.71	17.2/1.08	47.4/1.19
**MG_MID**	**3.61/0.91**	**19.9/0.93**	**70.8/0.80**
**Breast Cancer**			
MCF7	1.79/1.83	11.9/1.56	44.7/1.26
MDA-MB-231/ATCC	4.77/0.69	18.8/0.99	55.2/1.02
HS 578T	2.33/1.40	11.9/1.56	>100/0.57
BT-549	0.810/4.04	10.7/1.74	39.2/1.44
T-47D	3.58/0.91	19.7/0.94	61.2/0.92
MDA-MB-468	0.657/4.98	6.23/2.99	34.3/1.65
**MG_MID**	**2.32/1.41**	**13.2/1.41**	**55.8/1.01**
**MG_MID_60**	**3.27**	**18.6**	**56.5**

**Table 3:** IC_50_ values of the studied compounds and reference drug doxorubicin hydrochloride; nt—not tested.

Compound	Les-6418	Les-6416	Les-6381	Les-6423	Les-6424	DOX
Cell Line						
**Breast cancer**						
**MCF-7 24 h**	>20	4.70 ± 0.06	6.93 ± 0.13	>20	>20	2.46 ± 0.54
**MCF-7 48 h**	4.51 ± 0.04	2.09 ± 0.05	7.93 ± 0.10	>20	>20	0.81 ± 0.01
**MCF-7 72 h**	2.24 ± 0.07	1.29 ± 0.01	2.43 ± 0.12	>20	>20	0.26 ± 0.03
**MDA-MB-231 24 h**	>20	>20	>20	>20	>20	>20
**MDA-MB-231 48 h**	>20	>20	>20	>20	>20	3.91 ± 0.22
**MDA-MB-231 72 h**	>20	>20	>20	>20	>20	2.37 ± 0.15
**Colorectal cancer**						
**DLD-1 24 h**	>20	>20	18.23 ± 1.24	>20	>20	4.69 ± 0.48
**DLD-1 48 h**	>20	>20	3.86 ± 0.11	>20	>20	0.60 ± 0.08
**HT-29 24 h**	>20	>20	>20	>20	>20	>20
**HT-29 48 h**	7.77 ± 0.91	6.58 ± 0.37	4.58 ± 0.46	4.41 ± 0.35	>20	0.30 ± 0.04
**Gastric cancer**						
**AGS 24 h**	>20	>20	>20	>20	>20	>20
**AGS 48 h**	4.59 ± 0.42	3.45 ± 0.2	2.24 ± 0.31	14.47 ± 0.57	>20	0.39 ± 0.01
**Glioblastoma**						
**A172 24 h**	>20	>20	>20	>20	>20	>20
**A172 48 h**	>20	>20	8.65 ± 0.52	>20	>20	12.94 ± 0.89
**T98G 24 h**	16.87 ± 1.58	>20	13.11 ± 0.24	>20	>20	>20
**T98G 48 h**	>20	>20	4.09 ± 0.1	>20	>20	>20
**Normal cells**						
**MCF-10A 24 h**	>20	>20	8.37 ± 0.11	Nt	nt	>20
**MCF-10A 48 h**	7.24 ± 0.29	7.99 ± 0.48	0.97 ± 0.04	Nt	nt	0.16 ± 0.01
**MCF-10A 72 h**	6.66 ± 0.67	7.25 ± 0.3	2.03 ± 0.1	Nt	nt	0.11 ± 0.01
**C8-D1A 24 h**	12.86 ± 0.24	14.26 ± 1.52	nt	Nt	nt	>20
**C8-D1A 48 h**	7.42 ± 2.33	7.1 ± 0.73	nt	Nt	nt	0.25 ± 0.01
**Primary Dermal Fibroblast (HDFa) 24 h**	>20	>20	>20	>20	>20	>20
**Primary Dermal Fibroblast (HDFa) 48 h**	>20	>20	>20	>20	>20	>20
**Primary Dermal Fibroblast (HDFa) 72 h**	>20	>20	>20	>20	>20	0.99 ± 0.03

**Table 4:** ADMETLab 3.0 results for ADMET evaluation of the studied compounds. F_20%_, F_30%_, and F_50%_ signify the probability of bioavailability of the tested compounds to be bigger or smaller than 20, 30, and 50%, respectively. 1—less than, 0—equal or more than 20, 30, and 50%, respectively.

Name	Lipinski Rules [48]	Pfizer Rules [49]	Golden Triangle Rule [50]	GSK Rules [51]	F_20%_	F_30%_	F_50%_
Les-6416	+	+	+	−	0.092	0.275	0.945
Les-6418	+	+	+	−	0.107	0.639	0.956
Les-6381	+	+	+	−	0.377	0.375	0.965
Les-6423	+	+	+	+	0.765	0.977	0.966
Les-6424	+	+	+	+	0.804	0.966	0.967

**Table 5:** Molecular docking results for the studied compounds are displayed in kcal/mol.

Name	β-Tubulin		PPARγ	
	Docking Score	Ligand Efficiency	Docking Score	Ligand Efficiency
Ligand	−9.624	−0.341	−13.575	−0.309
6416	−9.876	−0.337	−8.913	−0.307
6418	−9.764	−0.418	−8.171	−0.282
6381	−8.764	−0.313	−8.116	−0.290
6423	−8.851	−0.354	−7.446	−0.298
6424	−8.408	−0.336	−7.312	−0.292

## Data Availability

Data are contained within the article.

## Supplementary Materials

The following supporting information can be downloaded at: https://www.mdpi.com/article/10.3390/cells14120861/s1, Figures S1–S16: Copies of 1H, 13C, NMR and LC-MS spectra of compounds 2, Les-6381, Les-6416, Les-6418, Les-6423 and Les-6424. Figures S17–S19: Protocols of DTP NCI anticancer screening of Les-6416 and Les-6418

## Abbreviations

ADMET	Absorption, distribution, metabolism, excretion and toxicity
AO	Acridine orange
DAPI	4′,6-Diamidino-2-phenylindole
DCF	2′,7′-Dichlorofluorescein
DOX	Doxorubicin hydrochloride
DTP	Developmental Therapeutic Program
EB	Ethidium bromide
EDDA	Ethylenediamine diacetate
ELISA	Enzyme-linked immunosorbent assay
FBS	Fetal bovine serum
FDA	The Food and Drug Administration
GI_50_	Molar concentration of the compound that inhibits 50% net cell growth
GP	Percentage of growth
IC_50_	Half maximal inhibitory concentration
LC_50_	Molar concentration of the compound that inhibits 50% net cell growth
MMP	Mitochondrial membrane potential
MTT	(3-(4,5-Dimethylthiazol-2-yl)-2,5-diphenyltetrazolium bromide) assay
NCI	National Cancer Institute
PBS	Phosphate-Buffered Saline
PDB	Protein Data Bank
PI	Propidium iodide
p53	Tumor protein P53
ROS	Reactive oxygen species
SRB	Sulforhodamine B
TCA	Trichloroacetic acid
TGI	Total growth inhibition
