# Supplementary material for: 5-Ene-2-arylaminothiazol-4(5H)-ones Induce Apoptosis in Breast Cancer Cells
Source: Cells. 2025 Jun 7;14(12):861. doi: 10.3390/cells14120861 (PMC12190436; doi:10.3390/cells14120861)
Supplement: Supplementary file 1 [file cells-14-00861-s001.zip › cells-3575749-supplementary.pdf]

Supplementary information

Supplementary data: The spectral and anticancer activity data of synthesized compounds

Table of Contents

Copies of ^{1}H, ^{13}C, NMR and LC-MS spectra of compounds **2, Les-6381, Les-6416, Les-6418, Les-6423 and Les-6424** …………………………….............. S1-S16

Protocols of DTP NCI anticancer screening of Les-6416 and Les-6418 …… S17-S19

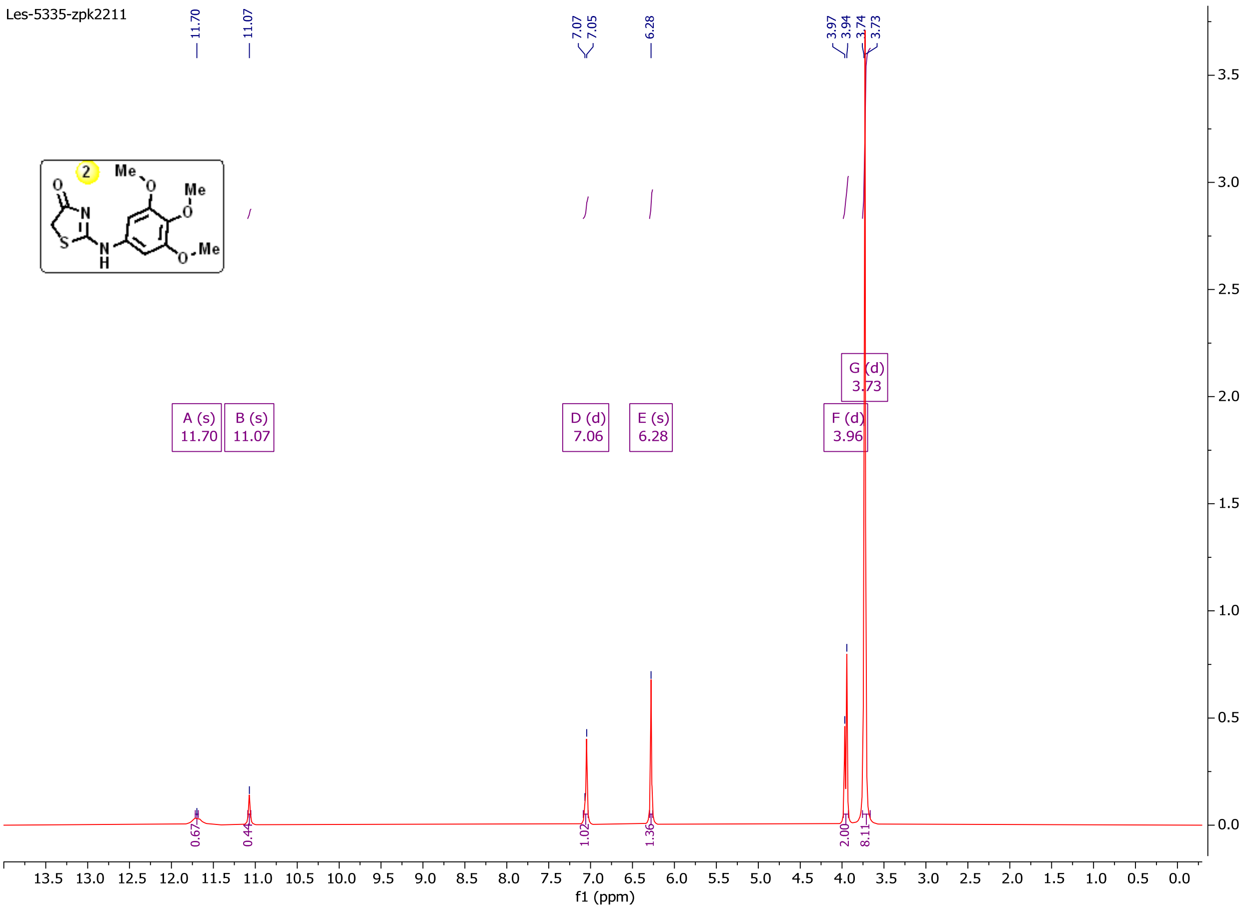

Figure S1. ^{1}H NMR spectrum of compound 2.

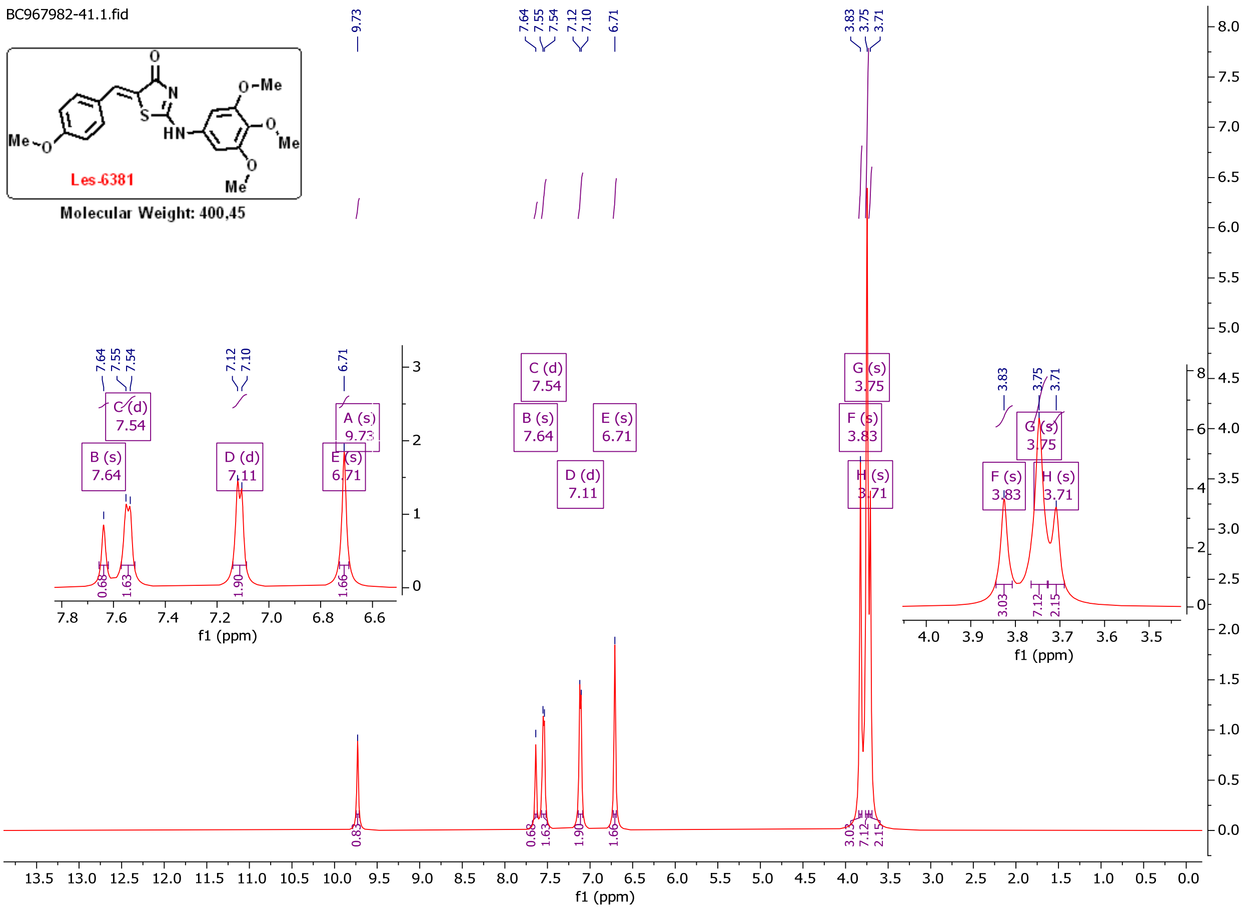

Figure S2. ^{1}H NMR spectrum of compound Les-6381.

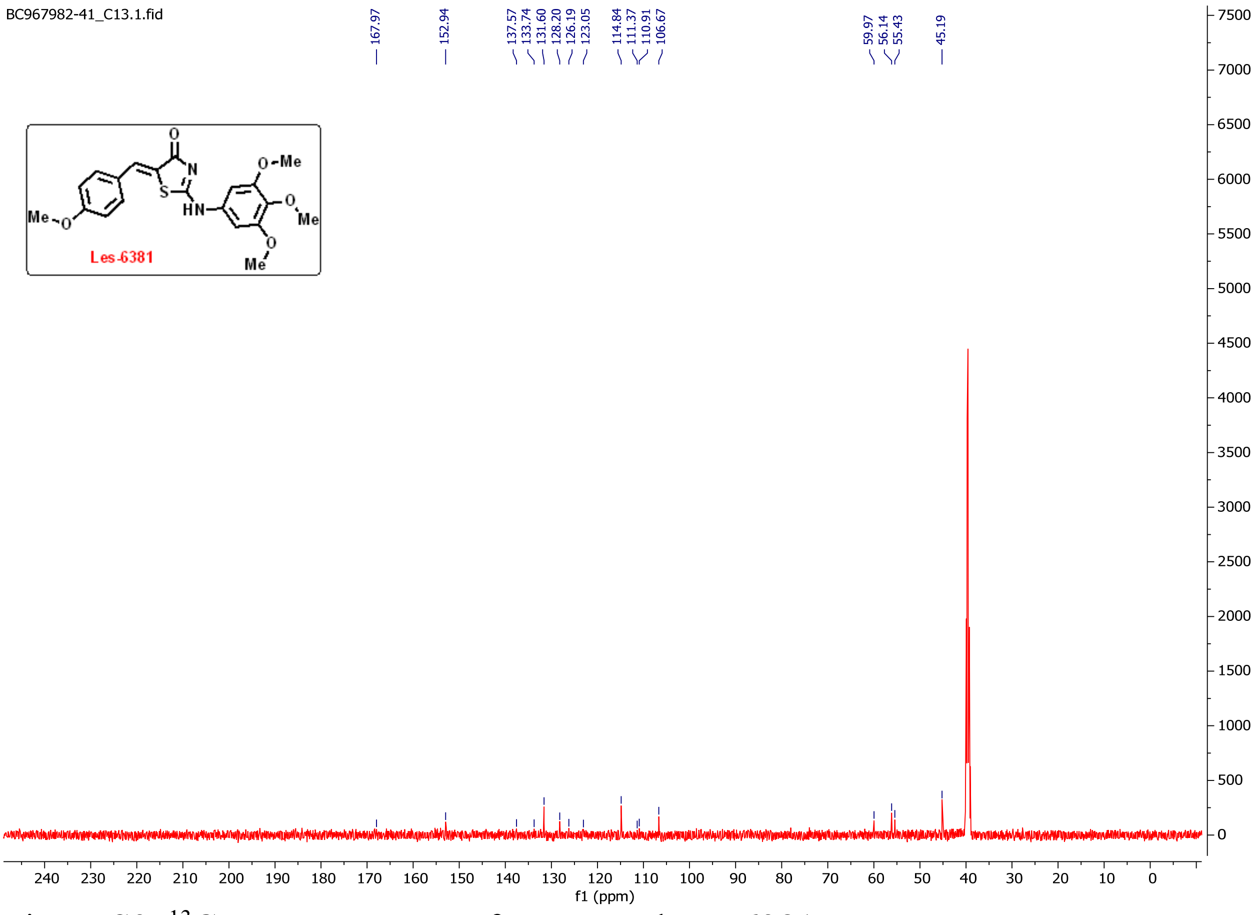

Figure S3. ^{13}C NMR spectrum of compound Les-6381.

#	RT	DAD1A	DAD1B	MSD1	MSD2	ELSD	MSD1 ions	MSD1 rt	MSD2 ions	MSD2 rt	Info
1	0.745	1.1%	---	2.0%	---	---	283.0(100)	0.751	---	---	
2	1.304	98.9%	100.0%	98.0%	84.8%	100.0%	401.2(100)	1.311	399.0(100)	1.310	
3	1.836	---	---	---	15.2%	---	---	---	458.2(100)	1.843	

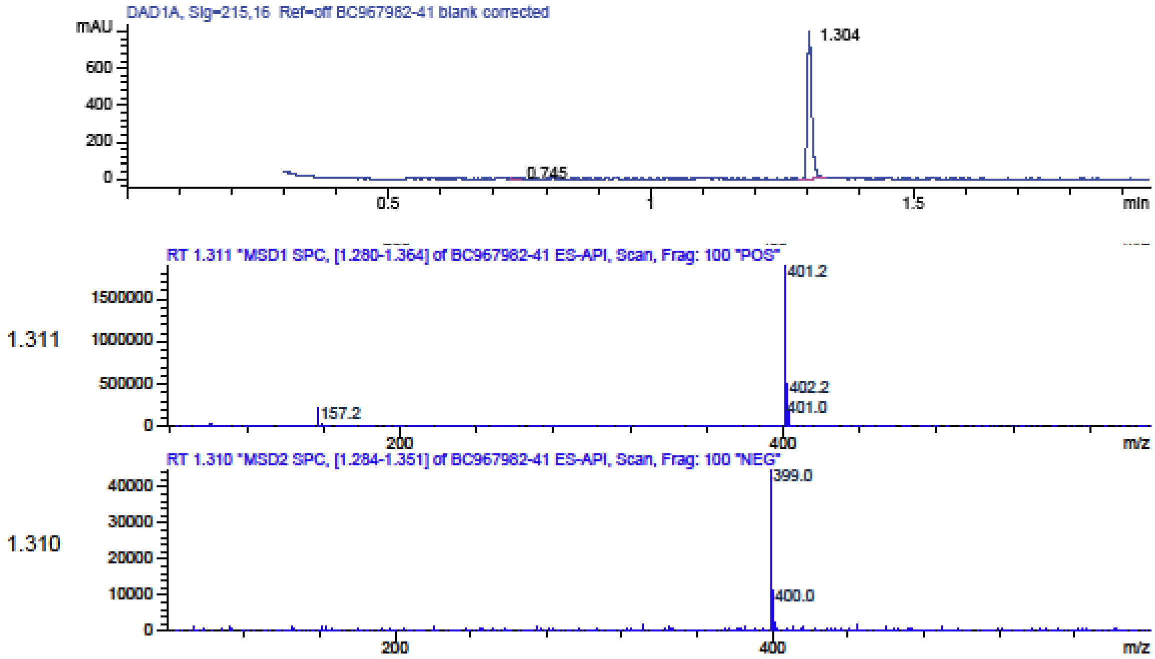

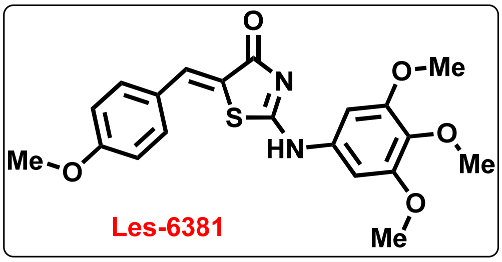

Molecular Weight: 400,45

Figure S4. LC-MS spectrum of compound Les-6381

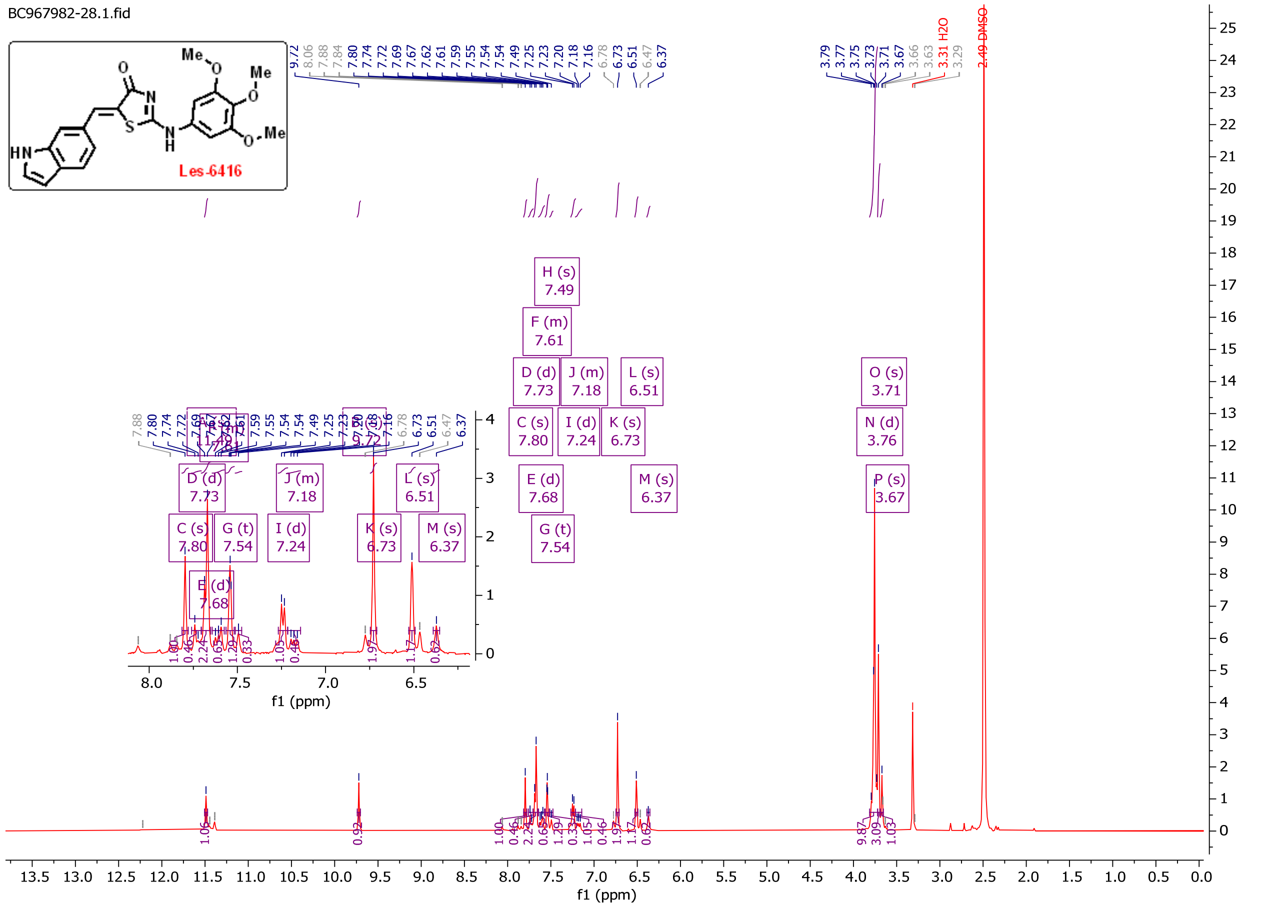

Figure S5. ^{1}H NMR spectrum of compound Les-6416.

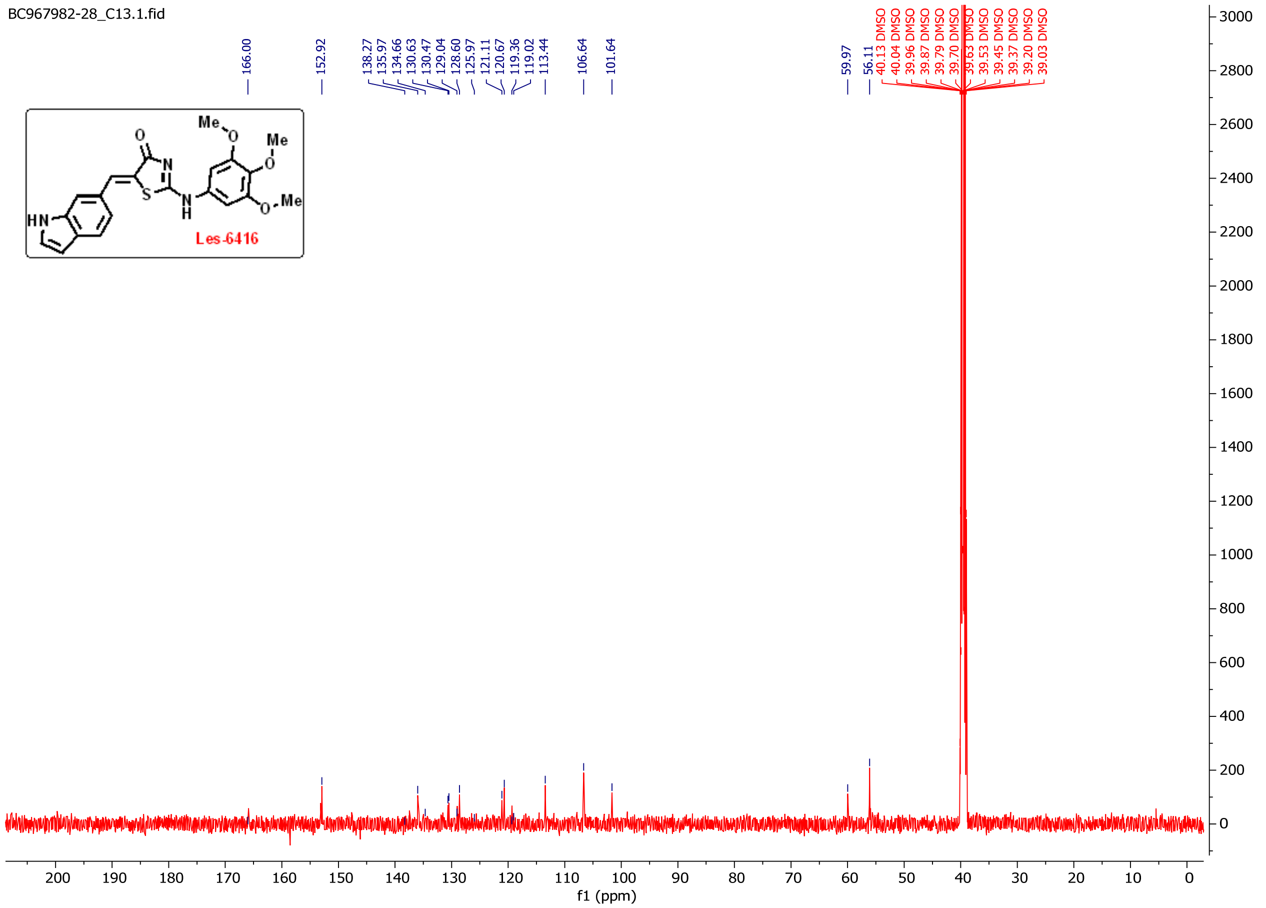

Figure S6. ^{13}C NMR spectrum of compound Les-6416.

#	RT	DAD1A	DAD1B	MSD1	MSD2	ELSD	MSD1 ions	MSD1 rt	MSD2 ions	MSD2 rt	Info
1	1.002	1.3%	0.7%	5.6%	---	---	323.2(100)	1.006	---	---	
2	1.260	73.0%	67.0%	94.4%	53.6%	79.8%	410.2(100)	1.267	408.0(100)	1.267	
3	1.297	25.7%	32.3%	---	46.4%	20.2%	---	---	408.0(100)	1.305	

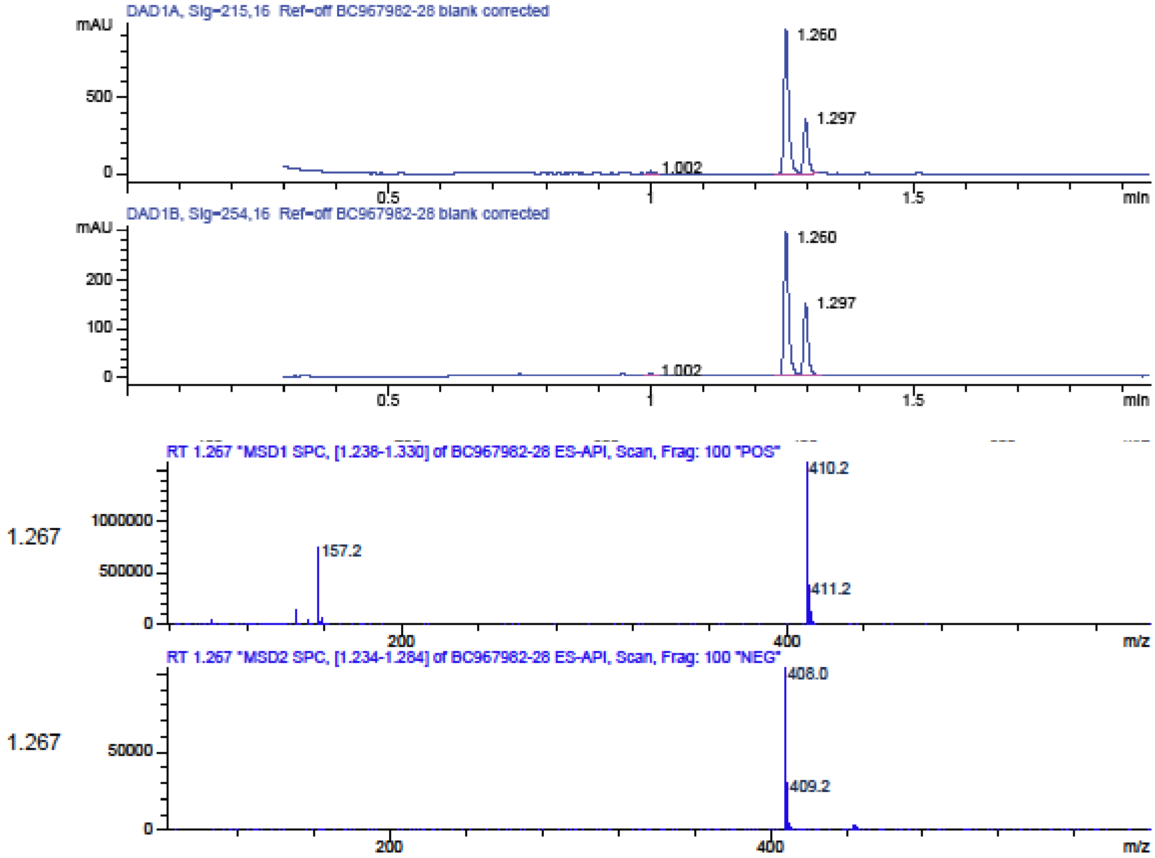

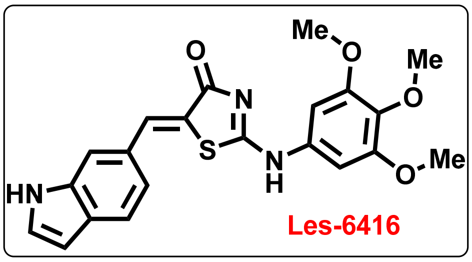

Molecular Weight: 409,46

Figure S7. LC-MS spectrum of compound Les-6416

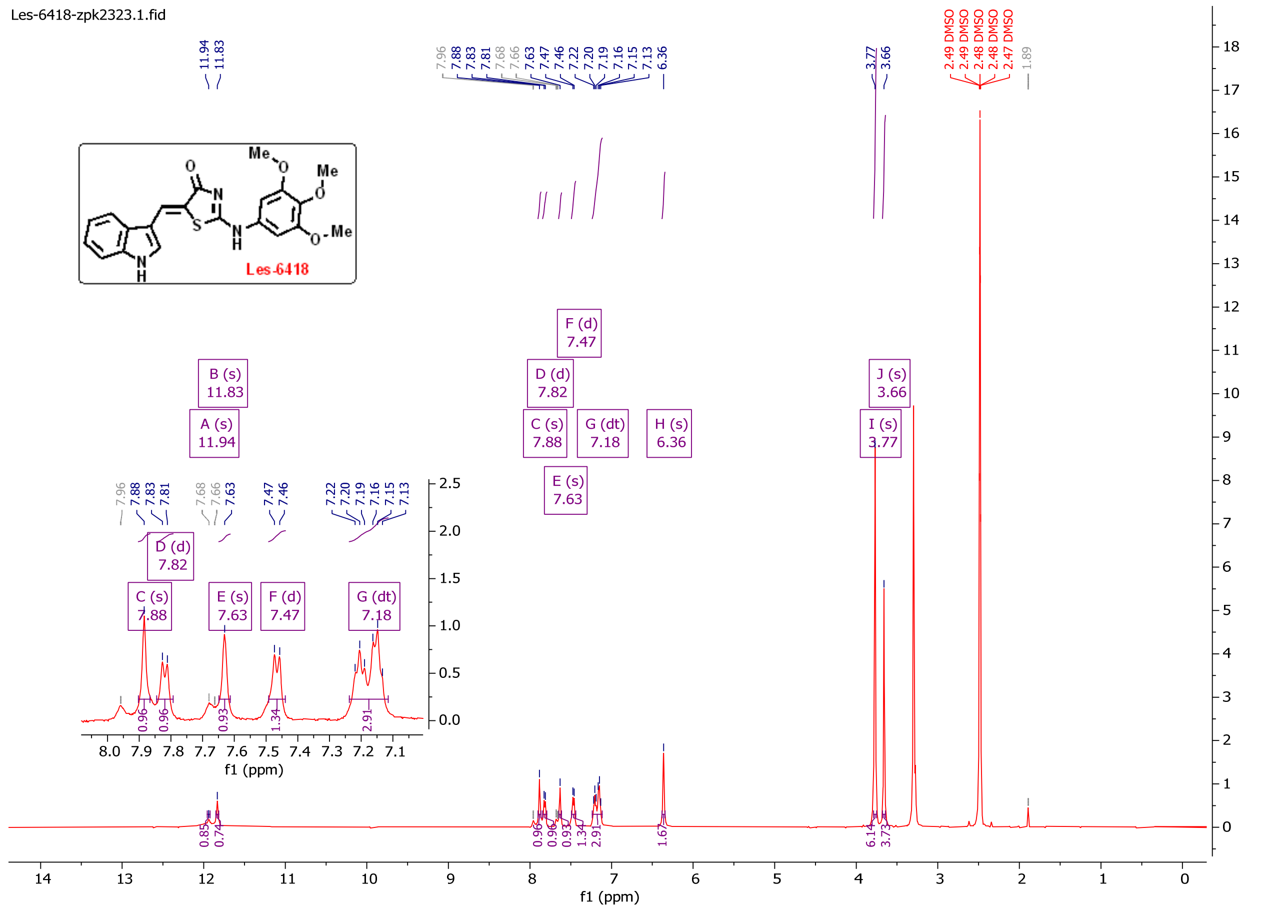

Figure S8. ^{1}H NMR spectrum of compound Les-6418.

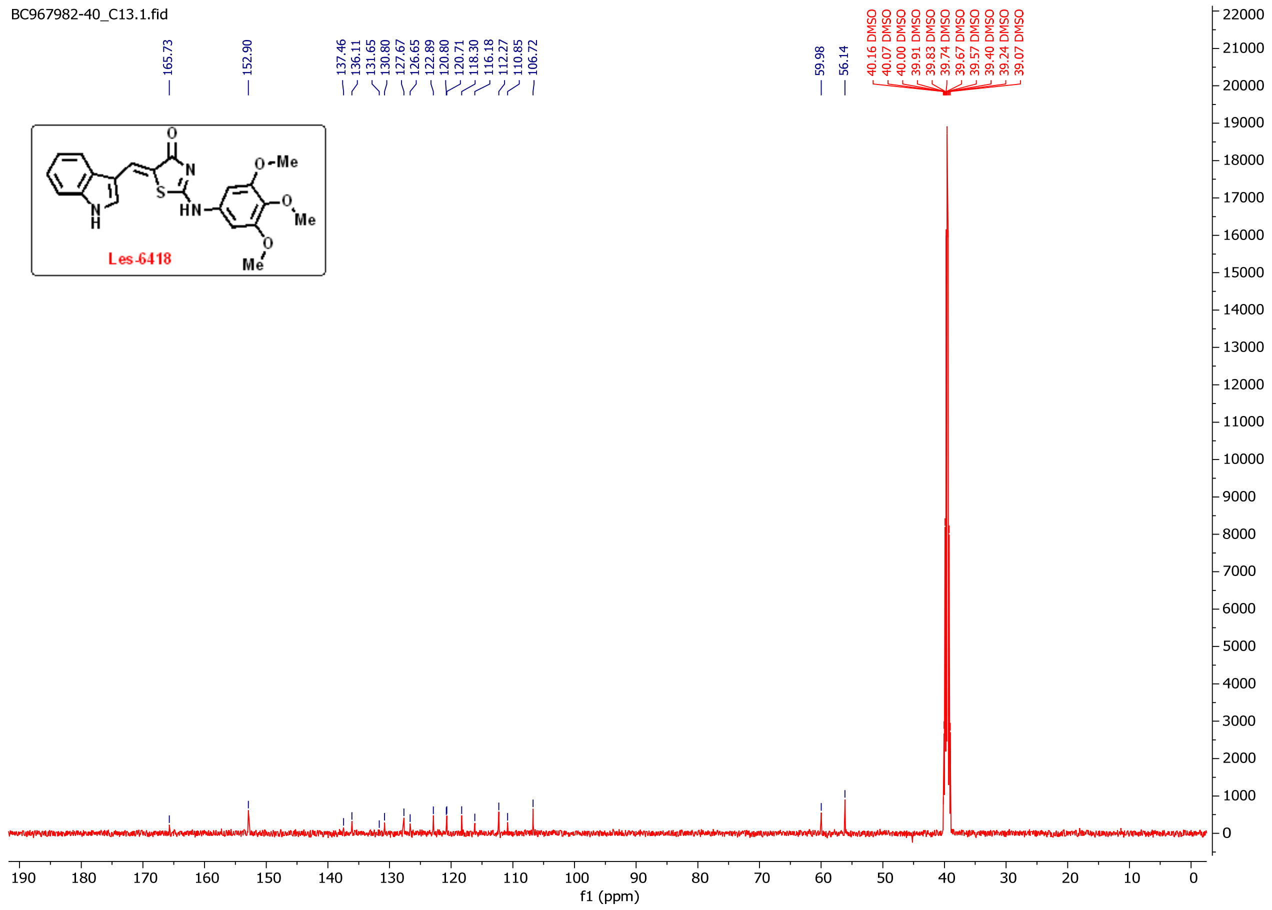

Figure S9. ^{13}C NMR spectrum of compound Les-6418.

MaxPeak: 100.00%
Ret_Time: 1.198 min

Mol Wt 0
Exact Mass

#	Time	Area%
1	1.198	100.00

RT 1.220

RT 1.218

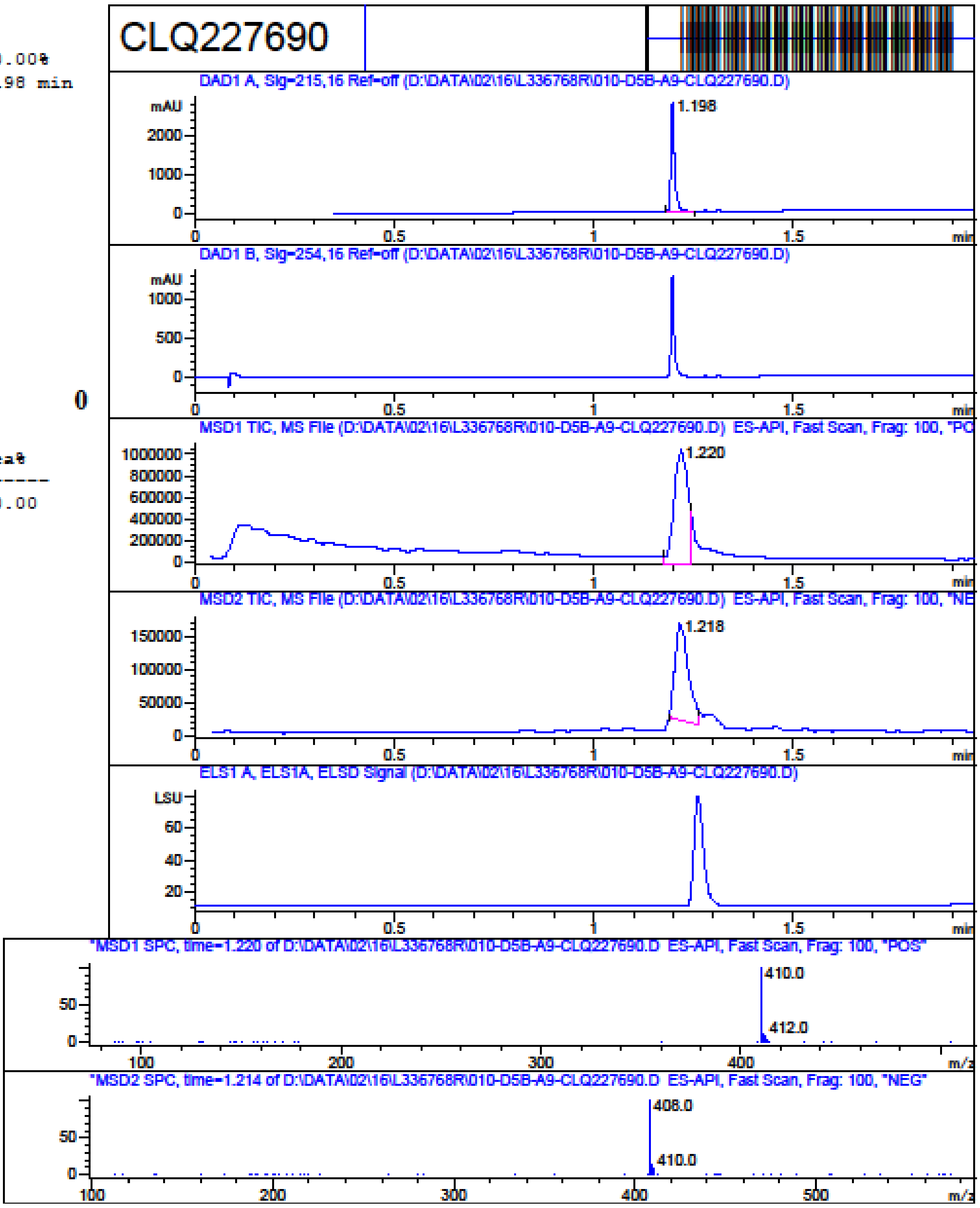

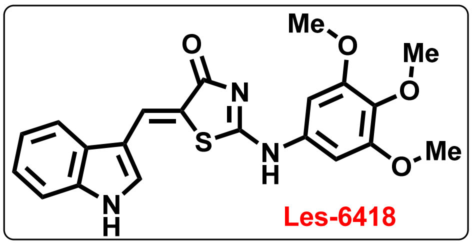

Molecular Weight: 409,46

Figure S10. LC-MS spectrum of compound Les-6418.

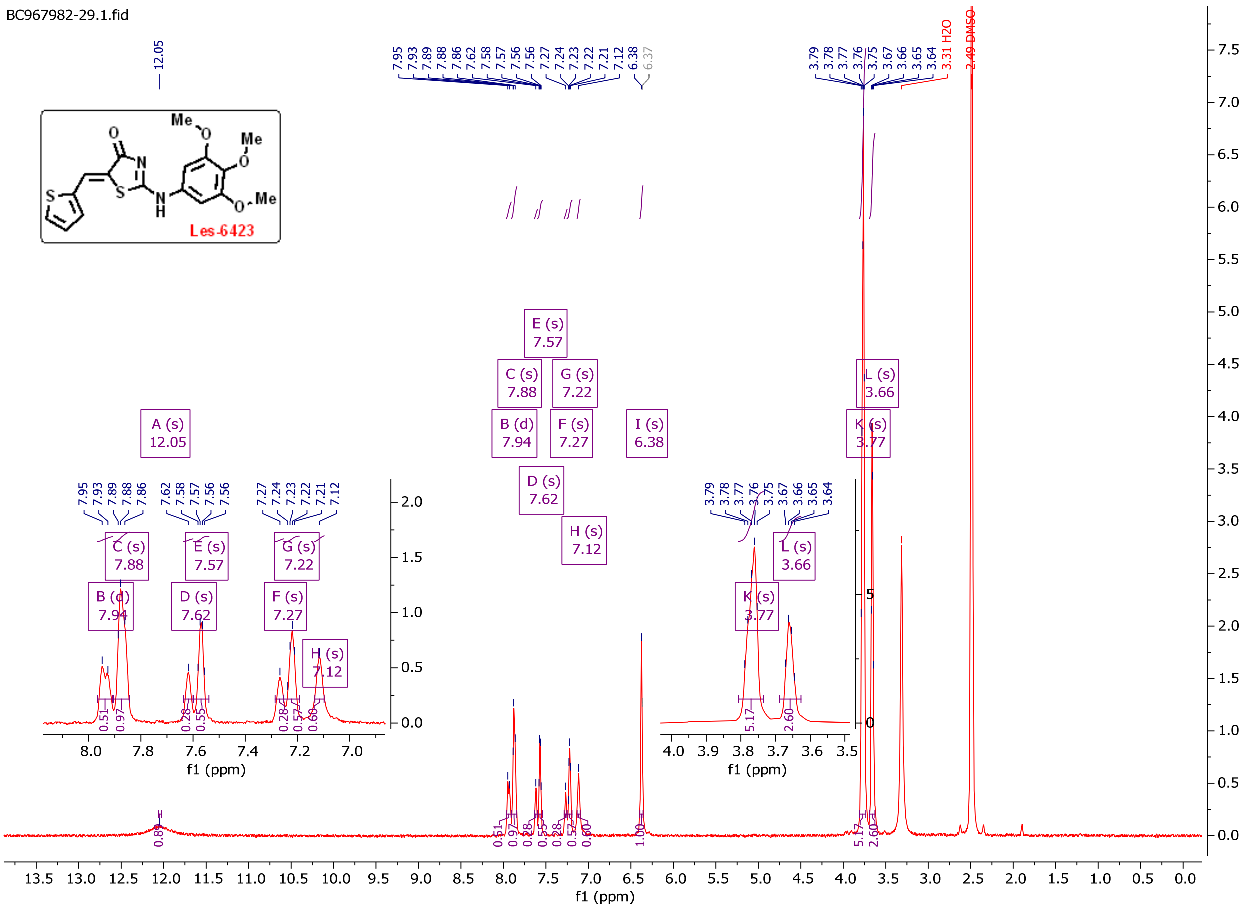

Figure S11. ^{1}H NMR spectrum of compound Les-6423.

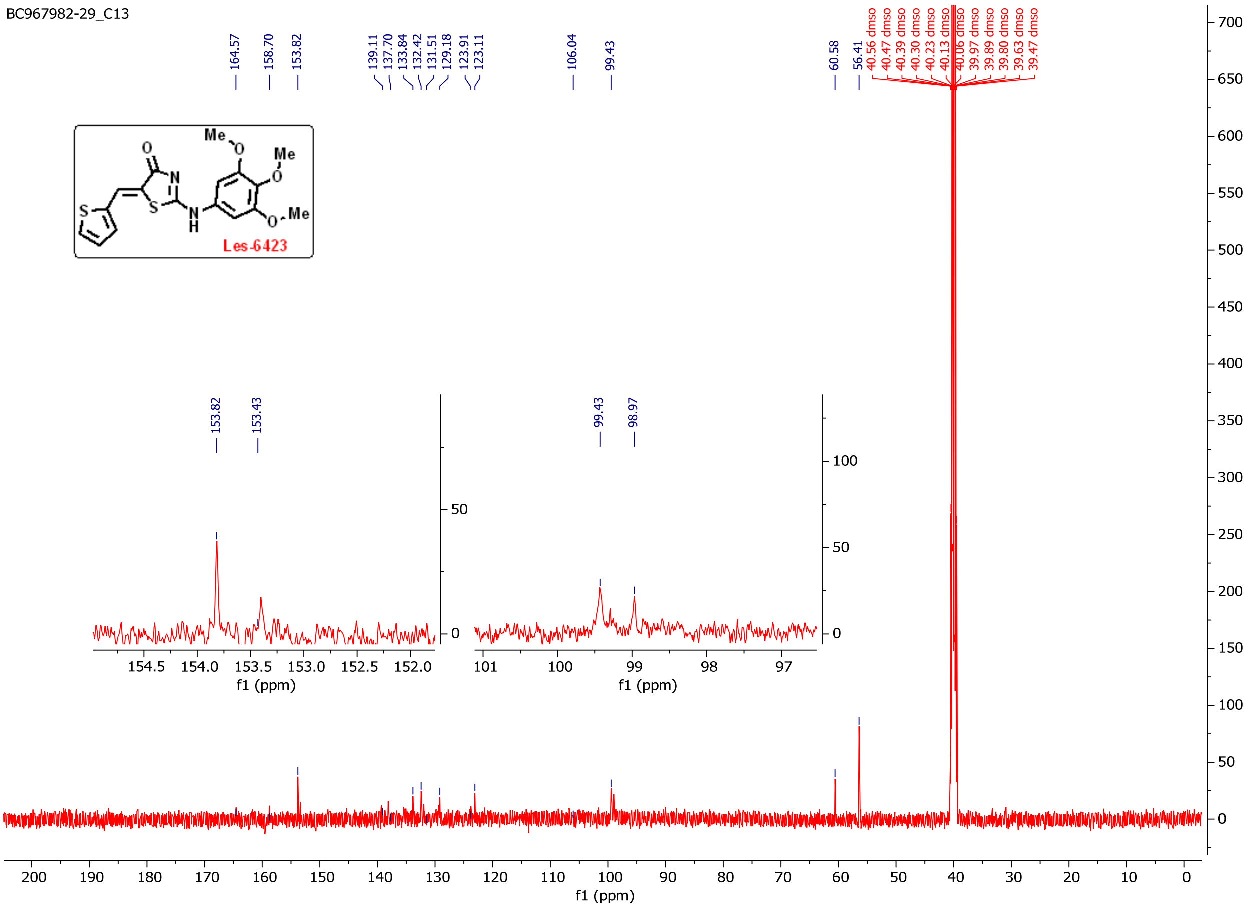

Figure S12. ^{13}C NMR spectrum of compound Les-6423.

#	RT	DAD1A	DAD1B	MSD1	MSD2	ELSD	MSD1 ions	MSD1 rt	MSD2 ions	MSD2 rt	Info
1	0.948	1.4%	0.9%	2.6%	---	---	283.0(100)	0.953	---	---	
2	1.294	98.6%	99.1%	97.4%	100.0%	100.0%	377.2(100)	1.302	375.0(100)	1.302	

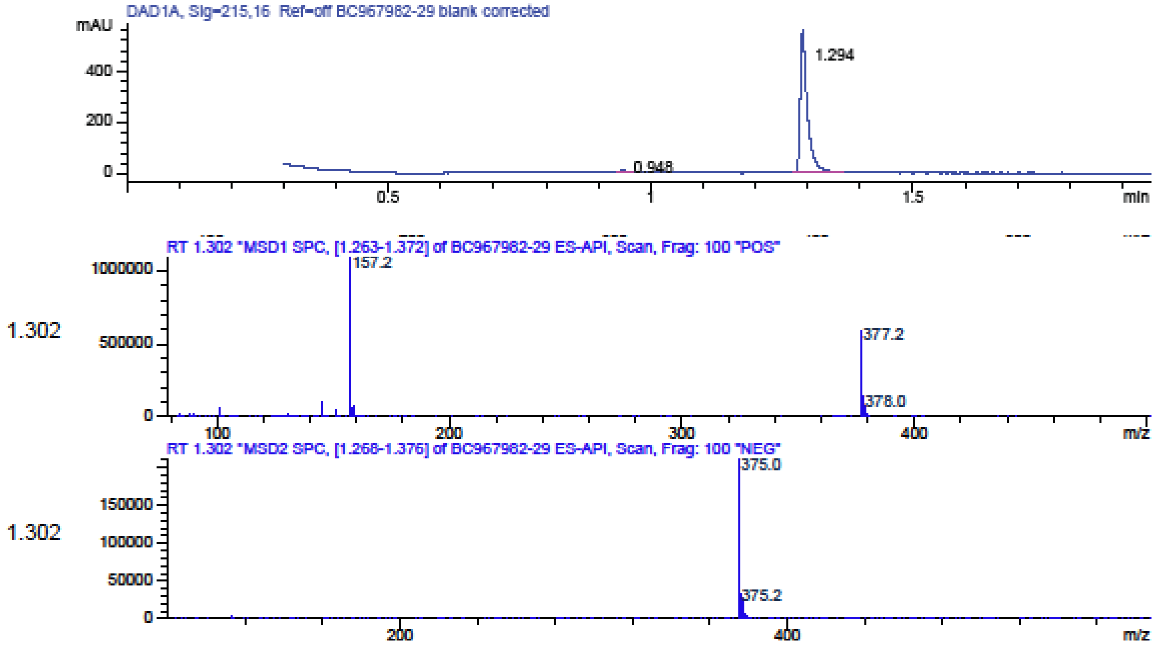

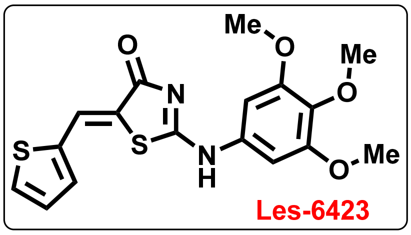

Molecular Weight: 376,45

Figure S13. LC-MS spectrum of compound Les-6423.

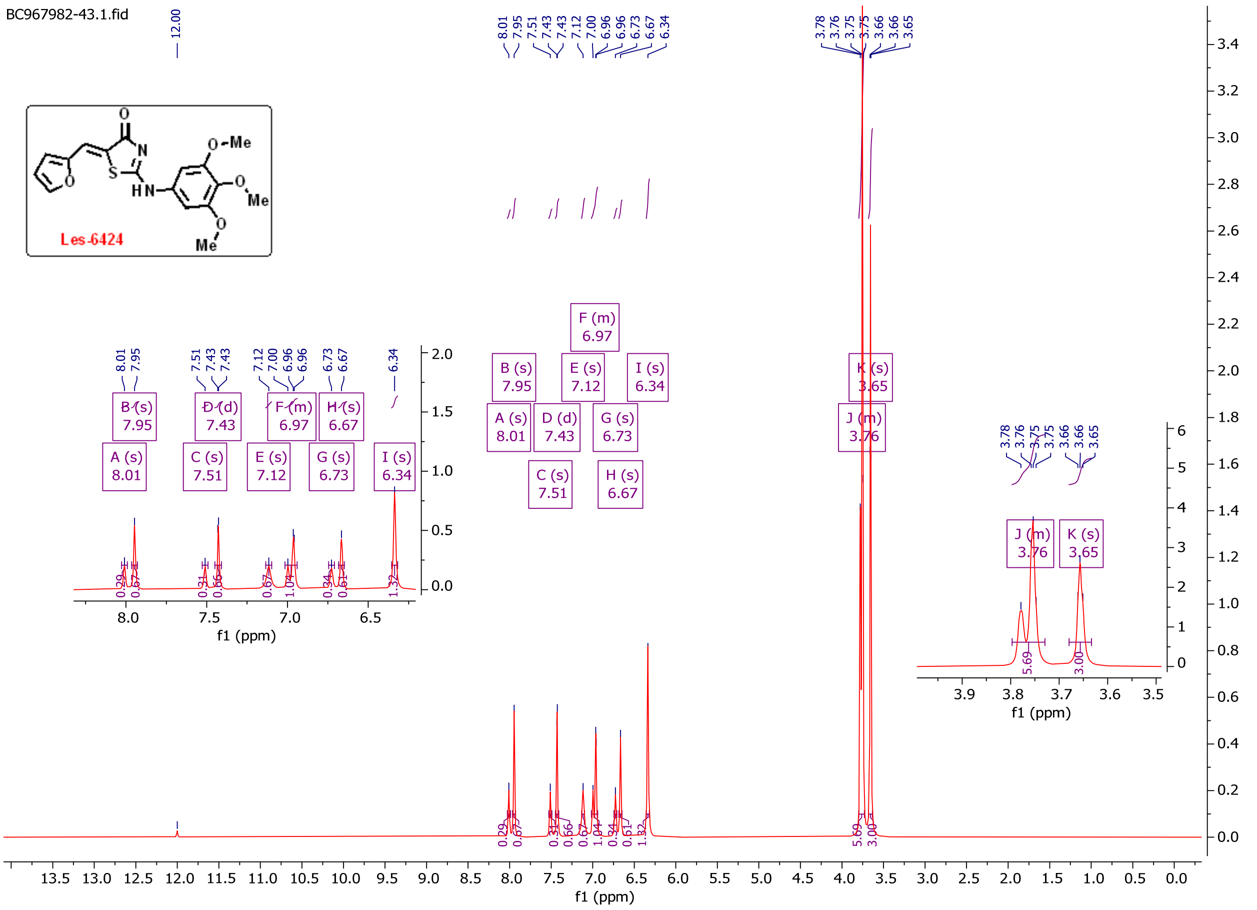

Figure S14. ^{1}H NMR spectrum of compound Les-6424.

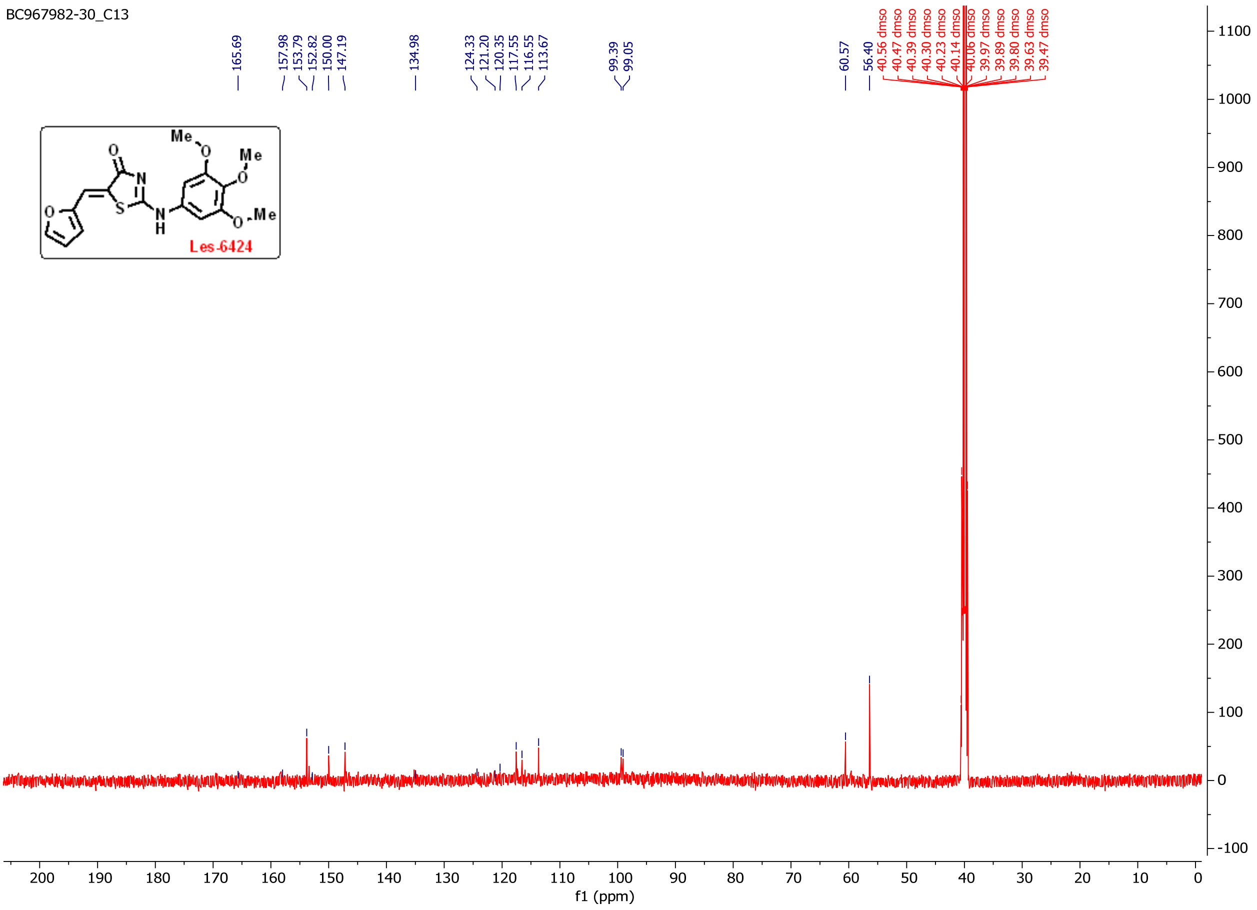

Figure S15. ^{13}C NMR spectrum of compound Les-6424.

#	RT	DAD1A	DAD1B	MSD1	MSD2	ELSD	MSD1 ions	MSD1 rt	MSD2 ions	MSD2 rt	Info
1	1.228	100.0%	100.0%	100.0%	100.0%	100.0%	361.0(100)	1.237	359.0(100)	1.236	

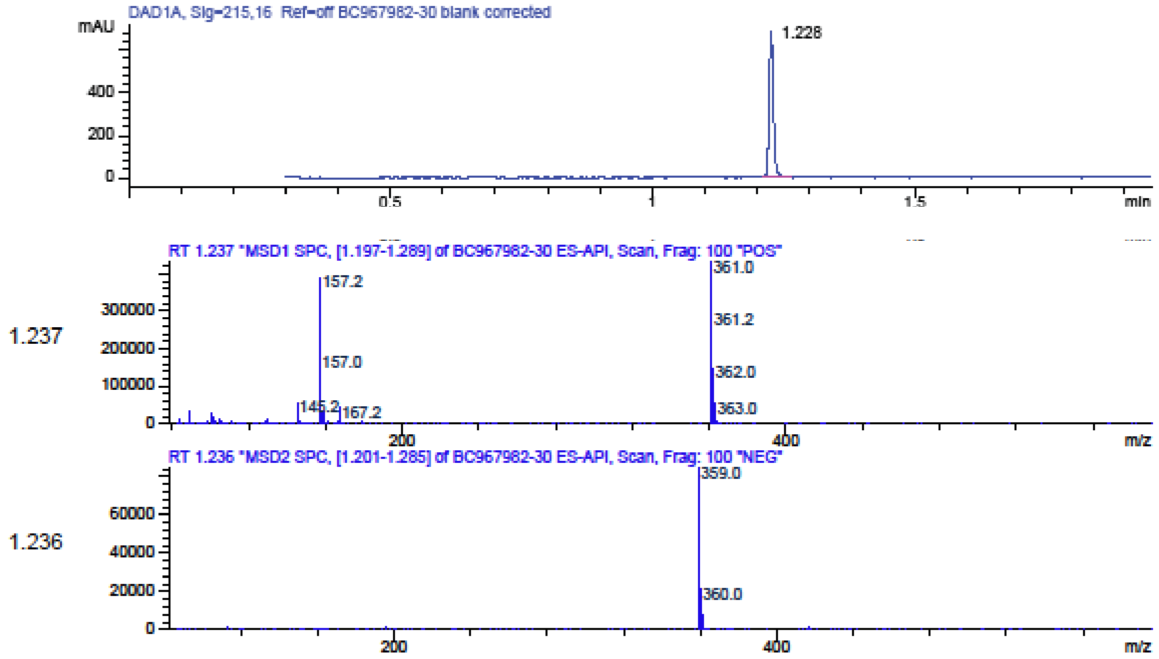

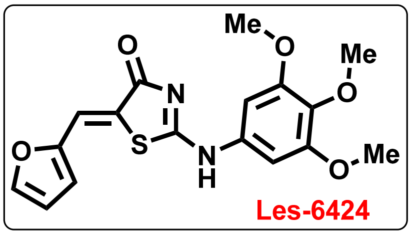

Molecular Weight: 360,38

Figure S16. LC-MS spectrum of compound Les-6424.

Developmental Therapeutics Program One Dose Mean Graph	NSC: D-839568 / 1	Conc: 1.00E-5 Molar	Test Date: Sep 12, 2022
	Experiment ID: 2209OS51		Report Date: Nov 20, 2024

Panel/Cell Line	Growth Percent
Leukemia	
CCRF-CEM	25.57
HL-60(TB)	15.10
K-562	25.35
MOLT-4	58.67
RPMI-8226	38.90
SR	30.11
Non-Small Cell Lung Cancer	
A549/ATCC	54.58
EKVX	49.60
HOP-62	53.38
HOP-92	88.76
NCI-H226	69.19
NCI-H23	60.00
NCI-H322M	55.66
NCI-H460	17.93
NCI-H522	3.77
Colon Cancer	
COLO 205	25.77
HCC-2998	58.11
HCT-116	56.11
HCT-15	24.42
HT29	7.66
KM12	40.04
SW-620	20.18
CNS Cancer	
SF-268	60.00
SF-295	5.01
SF-539	26.08
SNB-19	45.47
U251	31.60
Melanoma	
LOX IMVI	34.26
MALME-3M	33.70
M14	27.16
MDA-MB-435	-33.26
SK-MEL-2	21.62
SK-MEL-28	63.65
SK-MEL-5	-4.74
UACC-257	39.98
UACC-62	18.03
Ovarian Cancer	
IGROV1	44.25
OVCAR-3	23.17
OVCAR-4	74.60
OVCAR-5	70.91
OVCAR-8	65.82
SK-OV-3	47.91
Renal Cancer	
786-0	45.77
A498	97.05
ACHN	44.32
CAKI-1	35.86
RXF 393	46.25
SN12C	61.87
TK-10	75.68
UO-31	43.99
Prostate Cancer	
PC-3	37.18
DU-145	42.56
Breast Cancer	
MCF7	6.76
MDA-MB-231/ATCC	74.46
HS 578T	30.15
BT-549	38.13
T-47D	38.92
MDA-MB-468	1.33
Mean	39.56
Delta	72.82
Range	130.31

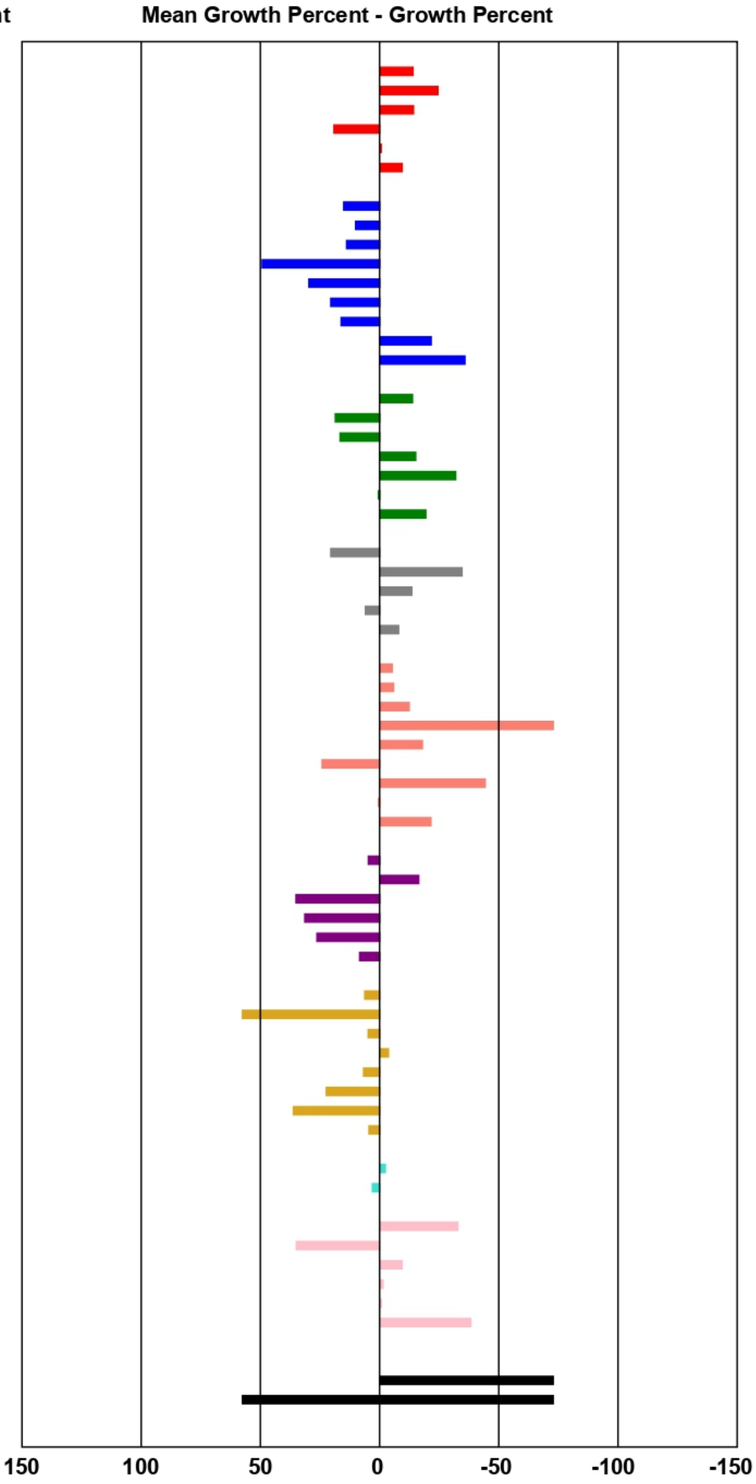

Figure S17. Protocol of anticancer screening data in concentration 10 μM for Les-6416.

Developmental Therapeutics Program One Dose Mean Graph	NSC: 832927 / 1	Conc: 1.00E-5 Molar	Test Date: Oct 18, 2021
	Experiment ID: 2110OS01		Report Date: Nov 20, 2024

Panel/Cell Line	Growth Percent
**Leukemia**	
CCRF-CEM	14.92
HL-60(TB)	-16.03
K-562	3.33
MOLT-4	2.95
RPMI-8226	-25.07
SR	2.60
**Non-Small Cell Lung Cancer**	
A549/ATCC	-69.42
EKVX	-41.83
HOP-62	-65.88
HOP-92	-72.26
NCI-H226	-46.47
NCI-H322M	-47.74
NCI-H460	-77.59
NCI-H522	-13.55
**Colon Cancer**	
COLO 205	-5.44
HCC-2998	-48.17
HCT-116	-59.21
HCT-15	-0.52
HT29	-50.75
KM12	-72.37
SW-620	-52.13
**CNS Cancer**	
SF-268	-38.18
SF-295	-73.38
SF-539	-85.30
SNB-19	-39.40
SNB-75	-63.76
U251	-79.79
**Melanoma**	
LOX IMVI	-61.47
MALME-3M	-30.08
M14	-18.72
MDA-MB-435	-77.11
SK-MEL-2	-70.64
SK-MEL-28	-34.45
SK-MEL-5	-34.71
UACC-257	4.51
UACC-62	-65.89
**Ovarian Cancer**	
IGROV1	-40.25
OVCAR-3	-76.96
OVCAR-4	-63.88
OVCAR-5	-52.54
OVCAR-8	-49.82
NCI/ADR-RES	11.45
SK-OV-3	-59.33
**Renal Cancer**	
786-0	-73.06
A498	-76.12
ACHN	-2.09
CAKI-1	-69.34
RXF 393	-69.16
SN12C	-88.00
TK-10	-35.04
UO-31	-48.87
**Prostate Cancer**	
PC-3	-71.87
DU-145	-57.56
**Breast Cancer**	
MCF7	-23.65
MDA-MB-231/ATCC	-40.74
HS 578T	-20.22
BT-549	-41.36
T-47D	2.48
MDA-MB-468	-19.88
Mean	-43.64
Delta	44.36
Range	102.92

Mean Growth Percent - Growth Percent

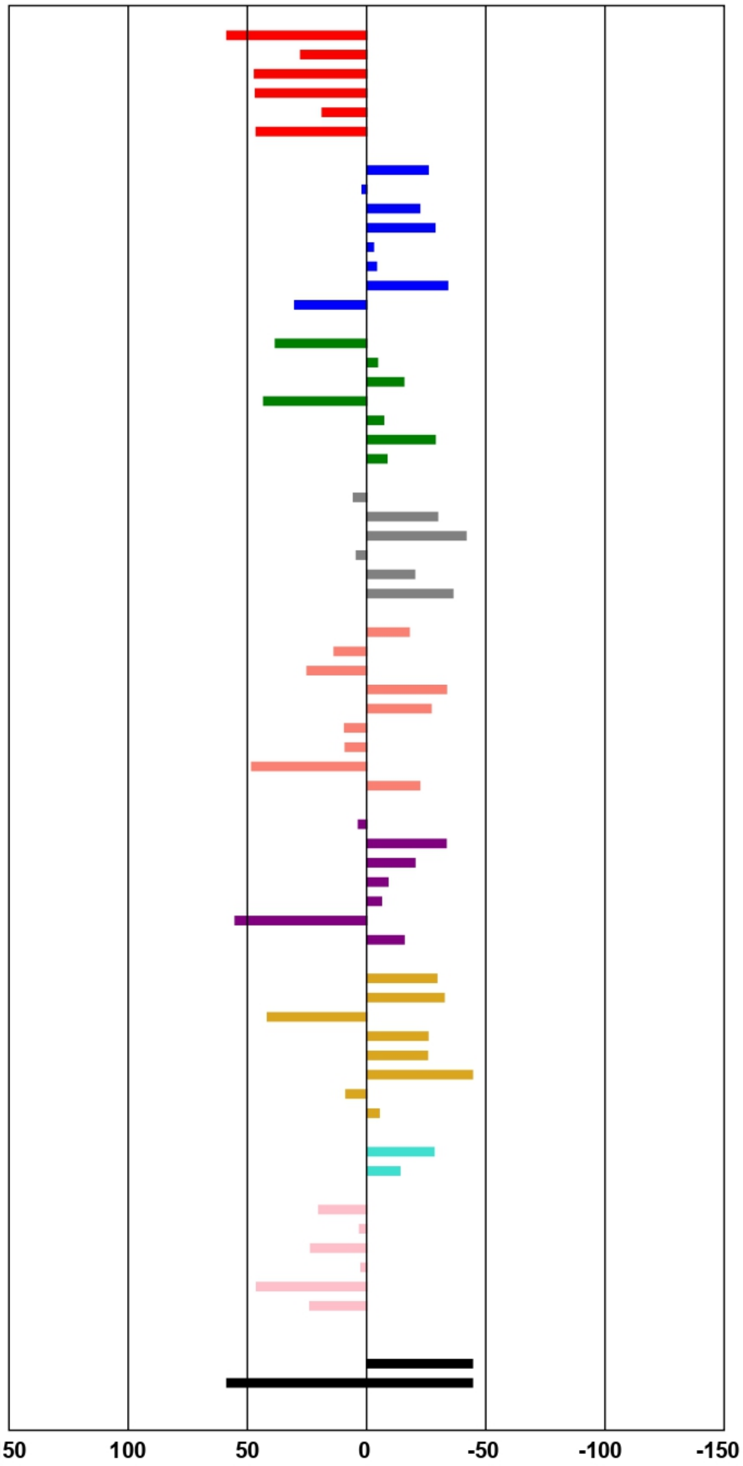

Figure S18. Protocol of anticancer screening data in concentration 10 μM for Les-6418.

National Cancer Institute Developmental Therapeutics Program
In-Vitro Testing Results

NSC : 832927 / 1	Experiment ID : 2112NS20	Test Type : 08	Units : Molar
Report Date : November 20, 2024	Test Date : December 06, 2021	QNS :	MC :
COMI : Les-6418	Stain Reagent : SRB Dual-Pass Related	SSPL : 0PZO	

			Log10 Concentration												
	Time		Mean Optical Densities					Percent Growth							
Panel/Cell Line	Zero	Ctrl	-8.0	-7.0	-6.0	-5.0	-4.0	-8.0	-7.0	-6.0	-5.0	-4.0	GI50	TGI	LC50
Leukemia															
CCRF-CEM	0.402	2.292	2.219	2.235	1.656	0.690	0.542	96	97	66	15	7	2.09E-6	> 1.00E-4	> 1.00E-4
HL-60(TB)	0.523	2.580	2.410	2.398	1.490	0.552	0.376	92	91	47	1	-28	8.55E-7	1.11E-5	> 1.00E-4
K-562	0.193	1.879	1.917	1.818	0.733	0.388	0.170	102	96	32	12	-12	5.26E-7	3.11E-5	> 1.00E-4
MOLT-4	0.524	2.323	2.338	2.462	1.823	0.871	0.382	101	108	72	19	-27	2.63E-6	2.60E-5	> 1.00E-4
RPMI-8226	0.758	2.810	2.777	2.783	2.671	1.501	0.808	98	99	93	36	2	5.73E-6	> 1.00E-4	> 1.00E-4
SR	0.415	2.027	1.913	1.780	0.841	0.644	0.350	93	85	26	14	-16	3.94E-7	2.99E-5	> 1.00E-4
Non-Small Cell Lung Cancer															
A549/ATCC	0.415	2.445	2.388	2.397	1.990	0.756	0.165	97	98	78	17	-60	2.84E-6	1.65E-5	7.36E-5
EKVX	0.717	1.626	1.655	1.585	1.549	1.132	0.100	103	96	91	46	-86	8.02E-6	2.22E-5	5.32E-5
HOP-62	0.562	1.716	1.641	1.627	1.438	0.743	0.200	93	92	76	16	-65	2.69E-6	1.57E-5	6.59E-5
HOP-92	1.188	1.924	1.886	1.797	1.770	0.961	0.372	95	83	79	-19	-69	1.98E-6	6.38E-6	4.20E-5
NCI-H226	0.788	1.328	1.279	1.283	1.221	0.897	0.287	91	92	80	20	-64	3.18E-6	1.74E-5	6.88E-5
NCI-H23	0.648	1.916	1.827	1.812	1.632	1.151	0.112	93	92	78	40	-83	5.33E-6	2.11E-5	5.40E-5
NCI-H322M	0.915	2.287	2.217	2.267	2.153	1.467	0.158	95	99	90	40	-83	6.38E-6	2.12E-5	5.42E-5
NCI-H460	0.312	3.064	3.140	3.105	2.217	0.575	0.149	103	101	69	10	-52	2.10E-6	1.43E-5	9.20E-5
NCI-H522	1.137	2.576	2.368	2.347	1.537	1.029	0.334	86	84	28	-9	-71	4.03E-7	5.56E-6	4.60E-5
Colon Cancer															
COLO 205	0.977	2.970	2.986	3.000	2.517	1.425	0.179	101	101	77	22	-82	3.14E-6	1.64E-5	4.96E-5
HCC-2998	0.594	1.870	1.785	1.852	1.587	1.145	0.036	93	99	78	43	-94	6.36E-6	2.07E-5	4.78E-5
HCT-116	0.297	2.491	2.412	2.456	1.440	0.308	0.086	96	98	52	1	-71	1.10E-6	1.02E-5	5.06E-5
HCT-15	0.280	1.640	1.650	1.638	1.144	0.414	0.017	101	100	64	10	-94	1.79E-6	1.24E-5	3.77E-5
HT29	0.343	2.211	2.280	2.327	1.239	0.348	0.090	104	106	48	0	-74	9.23E-7	1.01E-5	4.76E-5
KM12	0.807	3.215	3.197	3.096	2.000	1.241	0.306	99	95	50	18	-62	9.77E-7	1.68E-5	7.07E-5
SW-620	0.252	2.140	2.104	2.205	1.586	0.794	0.111	98	103	71	29	-56	3.10E-6	2.18E-5	8.50E-5
CNS Cancer															
SF-268	0.853	2.315	2.261	2.225	2.041	1.213	0.364	96	94	81	25	-57	3.56E-6	2.00E-5	8.13E-5
SF-295	0.671	2.697	2.677	2.562	2.098	0.935	0.068	99	93	70	13	-90	2.27E-6	1.34E-5	4.09E-5
SF-539	0.788	2.331	2.283	2.221	2.068	0.787	0.074	97	93	83	0	-91	2.49E-6	9.95E-6	3.55E-5
SNB-19	0.756	2.457	2.350	2.343	2.244	1.467	0.106	94	93	87	42	-86	6.60E-6	2.12E-5	5.22E-5
SNB-75	0.878	1.671	1.559	1.523	1.353	0.466	0.114	86	81	60	-47	-87	1.24E-6	3.63E-6	1.19E-5
U251	0.555	2.339	2.297	2.162	2.047	0.880	0.143	98	90	84	18	-74	3.27E-6	1.57E-5	5.47E-5
Melanoma															
LOX IMVI	0.405	2.720	2.734	2.605	2.223	0.757	0.035	101	95	79	15	-91	2.82E-6	1.39E-5	4.09E-5
MALME-3M	0.754	1.716	1.661	1.623	1.362	0.943	0.085	94	90	63	20	-89	2.01E-6	1.52E-5	4.39E-5
M14	0.530	1.951	1.949	1.929	1.437	0.792	0.141	100	98	64	18	-73	2.02E-6	1.59E-5	5.56E-5
MDA-MB-435	0.679	2.983	2.994	2.813	0.662	0.387	0.074	100	93	-3	-43	-89	2.80E-7	9.40E-7	1.42E-5
SK-MEL-2	1.404	2.933	2.930	2.907	2.294	1.523	0.236	100	98	58	8	-83	1.45E-6	1.22E-5	4.31E-5
SK-MEL-28	0.656	2.181	2.264	2.181	1.810	1.310	0.064	105	100	76	43	-90	6.07E-6	2.10E-5	4.99E-5
SK-MEL-5	0.951	3.158	3.138	3.131	2.521	1.328	0.037	99	99	71	17	-96	2.46E-6	1.41E-5	3.91E-5
UACC-257	0.750	1.976	1.928	1.802	1.653	1.247	0.114	96	86	74	41	-85	5.18E-6	2.11E-5	5.27E-5
UACC-62	0.944	3.114	3.092	3.044	2.581	1.766	0.088	99	97	75	38	-91	4.75E-6	1.97E-5	4.83E-5
Ovarian Cancer															
IGROV1	0.747	2.763	2.722	2.696	2.235	1.540	0.160	98	97	74	39	-79	4.91E-6	2.16E-5	5.72E-5
OVCAR-3	0.664	2.158	2.217	2.153	1.114	0.589	0.106	104	100	30	-11	-84	5.17E-7	5.32E-6	3.40E-5
OVCAR-4	0.769	1.972	2.044	2.014	1.856	0.799	0.052	106	103	90	2	-93	2.88E-6	1.06E-5	3.53E-5
OVCAR-5	0.641	1.884	1.922	1.859	1.717	1.030	0.033	103	98	87	31	-95	4.58E-6	1.77E-5	4.41E-5
OVCAR-8	0.934	3.263	3.258	3.257	3.165	2.166	0.292	100	100	96	53	-69	1.06E-5	2.72E-5	7.01E-5
NCI/ADR-RES	0.536	1.771	1.785	1.761	1.661	1.023	0.197	101	99	91	39	-63	6.24E-6	2.42E-5	7.42E-5
SK-OV-3	0.859	1.766	1.764	1.690	1.617	0.870	0.119	100	92	84	1	-86	2.56E-6	1.03E-5	3.85E-5
Renal Cancer															
786-0	0.525	2.174	2.065	2.008	1.912	0.619	0.163	93	90	84	6	-69	2.72E-6	1.19E-5	5.57E-5
A498	1.190	2.054	2.135	2.061	1.944	1.630	0.247	109	101	87	51	-79	1.02E-5	2.46E-5	5.96E-5
ACHN	0.484	1.955	1.958	1.953	1.845	0.607	0.018	100	100	93	8	-96	3.20E-6	1.20E-5	3.61E-5
CAKI-1	0.646	2.356	2.173	2.172	1.446	0.671	0.016	89	89	47	1	-98	8.40E-7	1.03E-5	3.31E-5
RXF 393	1.166	1.695	1.678	1.573	1.431	0.985	0.229	97	77	50	-16	-80	1.00E-6	5.80E-6	3.40E-5
SN12C	0.593	2.339	2.358	2.364	2.216	0.935	0.064	101	101	93	20	-89	3.85E-6	1.51E-5	4.36E-5
TK-10	1.365	2.329	2.376	2.402	2.337	1.812	0.232	105	108	101	46	-83	8.56E-6	2.28E-5	5.56E-5
UO-31	0.834	2.953	2.848	2.855	2.744	1.154	0.038	95	95	90	15	-95	3.43E-6	1.37E-5	3.88E-5
Prostate Cancer															
PC-3	0.617	2.304	2.261	2.235	1.716	1.099	0.296	97	96	65	29	-52	2.59E-6	2.26E-5	9.42E-5
DU-145	0.467	1.777	1.828	1.815	1.732	0.817	0.062	104	103	97	27	-87	4.63E-6	1.72E-5	4.74E-5
Breast Cancer															
MCF7	0.316	1.780	1.724	1.722	1.262	0.414	0.062	96	96	65	7	-81	1.79E-6	1.19E-5	4.47E-5
MDA-MB-231/ATCC	0.619	1.773	1.824	1.757	1.698	0.958	0.139	104	99	94	29	-78	4.77E-6	1.88E-5	5.52E-5
HS 578T	1.452	2.783	2.719	2.670	2.482	1.488	0.975	95	91	77	3	-33	2.33E-6	1.19E-5	> 1.00E-4
BT-549	1.346	2.418	2.391	2.315	1.838	1.374	0.189	98	90	46	3	-86	8.10E-7	1.07E-5	3.92E-5
T-47D	0.902	2.326	2.239	2.171	1.969	1.329	0.256	94	89	75	30	-72	3.58E-6	1.97E-5	6.12E-5
MDA-MB-468	0.890	1.569	1.534	1.497	1.170	0.795	0.142	95	89	41	-11	-84	6.57E-7	6.23E-6	3.43E-5

Figure S19. Protocol of in depth anticancer screening data of Les-6418 at concentrations ranging from 10^{-4} to 10^{-8} M.
